# Supplementary material for: Timing of daily calorie loading affects appetite and hunger responses without changes in energy metabolism in healthy subjects with obesity
Source: Cell Metab. 2022 Oct 4;34(10):1472–1485.e6. doi: 10.1016/j.cmet.2022.08.001 (PMC9605877; doi:10.1016/j.cmet.2022.08.001)
Supplement: Document S1. Figures S1–S3 and Tables S1–S6 [file mmc1.pdf]

Cell Metabolism, Volume *34*

Supplemental information

Timing of daily calorie loading affects appetite and hunger responses without changes in energy metabolism in healthy subjects with obesity

Leonie C. Ruddick-Collins, Peter J. Morgan, Claire L. Fyfe, Joao A.N. Filipe, Graham W. Horgan, Klaas R. Westerterp, Jonathan D. Johnston, and Alexandra M. Johnstone

Supplemental Figure 1:

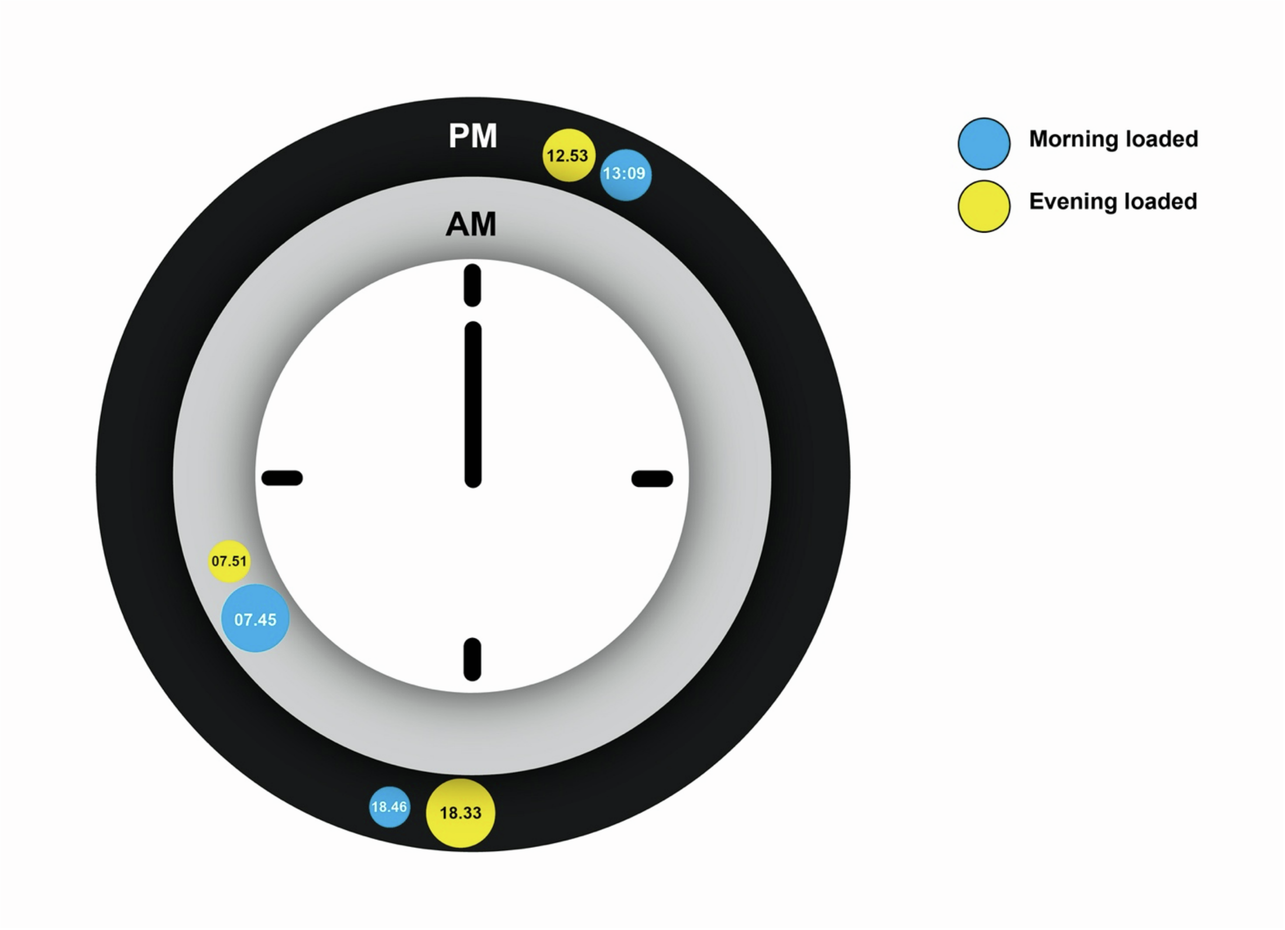

Supplemental Figure 1: Average recorded subjects' mealtime for the meals on the morning loaded (ML, blue circles) and evening loaded (EL, yellow) diets, where the size of the circle reflects the size of the meal, related to Figure 1.

Supplemental Figure 2:

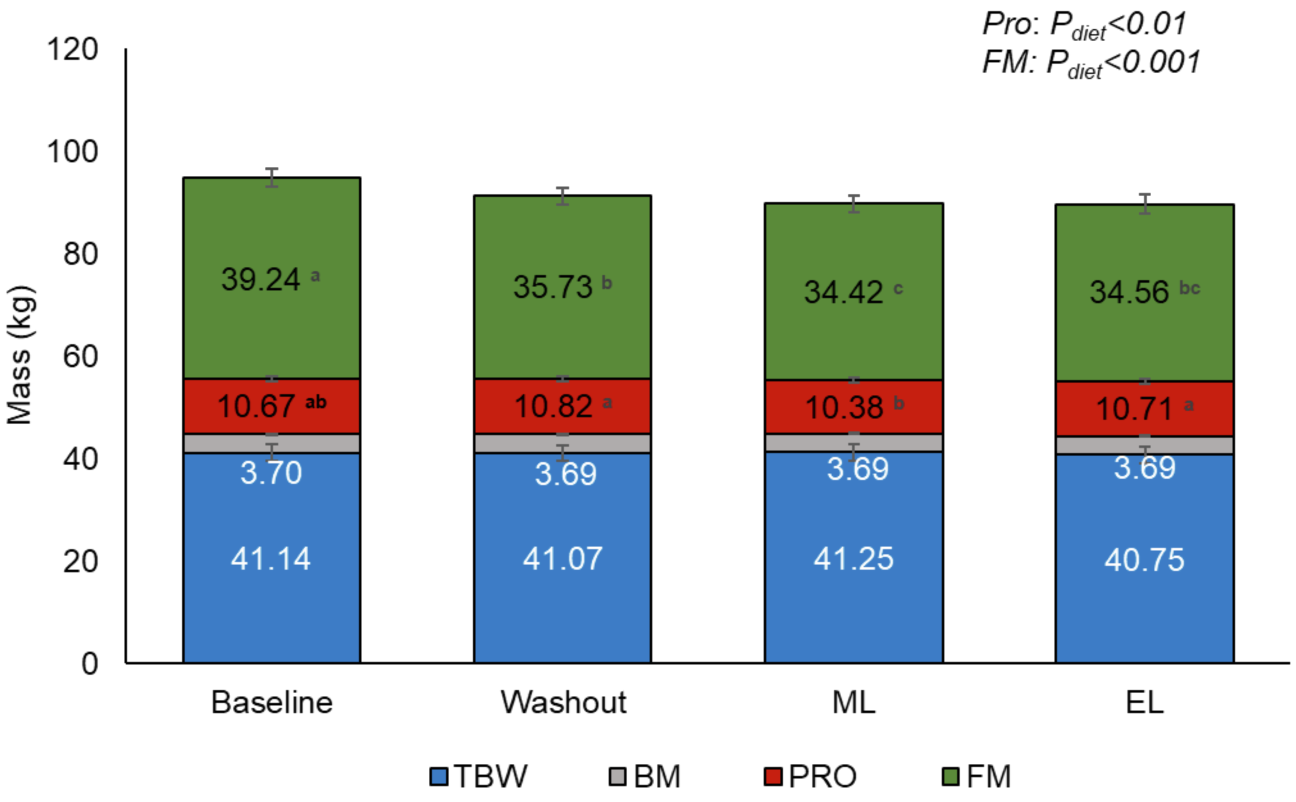

Supplemental Figure 2: Body composition measured with the 4-compartment model, related to Figure 2. Body volume (densitometry) derived from the BODPOD, total body mineral derived from dual energy x-ray absorptiometry (DXA) and total body water (TBW) from deuterium isotope dilution. Participants had significantly lower body weight following both the ML and EL diets compared to B and W, but with no difference between the two weight loss diets. FM was significantly lower at the end of the ML and EL diets compared to B, but there was no significant difference between the weight loss diets. There were no significant differences in bone mineral or TBW between any of the diets. Protein mass was significantly lower after the ML diet compared to EL diet and W, but it was not lower than B.

B: baseline, BM: bone mineral, EL: evening loaded, FM: fat mass, ML: morning loaded, PRO: protein mass, W: washout

Supplemental Figure 3:

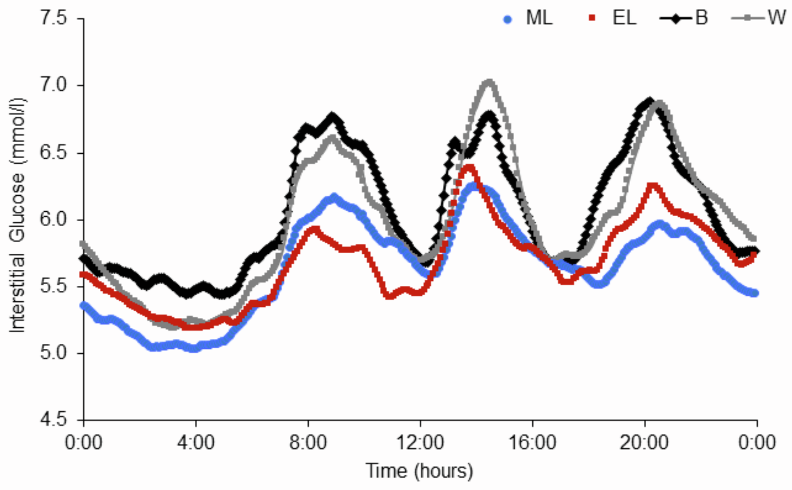

Supplemental Figure 3: Group mean continuous glucose monitoring for all diet periods, related to Figure 5

Supplemental Table 1: Pre-intervention characteristics of overweight adults who completed the dietary protocol, related to Figure 4.

	Males (n = 16)		Females (n = 14)		All (n = 30)	
Age (y)	51.0 ± 3.0	(27 - 70)	50.8 ± 3.2	(29 - 67)	50.9 ± 2.1	(27 - 70)
Height (m)	1.78 ± 0.01	(1.67 - 1.90)	1.63 ± 0.02	(1.50 - 1.73)	1.71 ± 0.02	(1.50 - 1.90)
Weight (kg)	105.6 ± 3.7	(86.1 - 135.3)	83.7 ± 2.6	(73.6 - 109.0)	95.4 ± 3.1	(73.6 - 135.3)
BMI (kg/m^2)	33.5 ± 1.2	(27.3 - 41.8)	31.3 ± 0.7	(28.0 - 38.6)	32.5 ± 0.7	(27.3- 41.8)
RMR (kcal)	2112 ± 71	(1814 – 2894)	1534 ± 48	(1153 – 1881)	1842 ± 69	(1153 – 2894)
SBP (mmHg)	138 ± 3	(111 – 165)	128 ± 4	(103 – 156)	133 ± 3	(103 – 165)
DBP (mmHg)	88 ± 2	(75 – 104)	84 ± 2	(70 – 97)	86 ± 2	(70 – 104)
Pulse (beats/min)	62 ± 2	(51 – 78)	65 ± 3	(52 – 82)	63 ± 2	(51 – 82)
HbA1c (mmol/mol)	36.1 ± 1.0	(29 – 42)	36.3 ± 1.0	(29 – 45)	36.2 ± 0.7	(29 – 45)
Chronotype (MEQ)[a]	3.8 ± 0.3	(2 – 5)	3.6 ± 0.3	(2 – 5)	3.7 ± 0.2	(2 – 5)
PSQI score[b]	5.3 ± 0.8	(1 – 12)	5.2 ± 0.7	(2 – 11)	5.2 ± 0.5	(1 – 12)

Data are expressed as mean ± SEM; range in parentheses

[a]MEQ: Morning -Evening questionnaire (Horne and Östberg, 1976) Chronotype type range: 1 – 5. (1 = definitely evening to 5 = definitely morning).

[b]PSQI: Pittsburgh sleep quality index (Buysse et al., 1989). Range: 0–28. Score ≤14 normal >14 indicative of insomnia.

DBP: Diastolic blood pressure, EL: evening loaded, ML: morning loaded, RMR: resting metabolic rate, SBP: Systolic blood pressure

Supplemental Table 2: Macronutrient composition of the diets consumed during the study dietary periods, as described in the STAR methods section.

Diet	Energy (kcal/d)	Fat (%)	Protein (%)	CHO (%)	Alcohol (%)	Fiber (%)
Baseline	2658[a]	30.0[b]	15.1 [b]	53.0[b]	0.2[b]	1.8[b]
Washout	2472[c]	30.0[b]	15.1 [b]	53.0[b]	0.1[b]	1.8[b]
ML	1736[d]	34.7[a]	29.7[c]	34.2[c]	0.1[b]	1.3[c]
EL	1749[d]	34.8[a]	29.8[c]	34.0[c]	0.1[b]	1.4[c]
SED	72.7	0.58	0.39	0.74	0.61	0.044
P	< 0.001	< 0.001	< 0.001	< 0.001	< 0.001	< 0.001

Data are expressed as means, n=30.

Analyzed for diet effect by ANOVA, means in the same row not sharing a superscript are significantly different (P<0.05). SED is based on within-volunteer spread.

CHO: carbohydrate, EL: evening loaded, ML: morning loaded

Supplemental Table 3: Meal and Sleep times throughout the study dietary periods, as decribed in the STAR methods section.

	Baseline	Washout	ML	EL	SED	P
Mealtime						
Breakfast (HH:MM)	08:07[a]	07:46[b]	07:43[b]	07:52[ab]	00:07	0.011
Lunch (HH:MM)	13:00	13:06	13:10	12:58	00:07	0.251
Dinner (HH:MM)	18:27[a]	18:44[ab]	18:51[b]	18:32[a]	00:06	0.002
Duration of feeding (HH:MM)	10:20[a]	10:56[bc]	11:05[b]	10:39[ac]	00:09	< 0.001
Sleep parameters						
Wake time (HH:MM)	06:00	06:07	06:03	06:04	00:05	0.675
Arise time (HH:MM)	06:14	06:26	06:21	06:26	00:06	0.201
Bed time (HH:MM)	21:57[a]	22:09[ab]	22:16[b]	22:13[b]	00:06	0.016
Sleep time (HH:MM)	22:16[a]	22:35[b]	22:34[b]	22:32[b]	00:05	0.005
Sleep latency	11:87[a]	8.34[b]	7.68[b]	9.96[ab]	0.089	< 0.001
Sleep disturbances	1.23[a]	0.72[b]	0.64[b]	0.69[b]	0.046	< 0.001
Sleep quality	4.56	4.11	4.28	4.15	0.21	0.13
Attempted sleep duration (HH:MM)	07:43	07:32	07:30	07:32	00:07	0.265
Actual sleep duration (HH:MM)	07:06	07:08	07:08	07:02	00:08	0.888

Analyzed for diet effect by ANOVA, means in the same row not sharing a superscript are significantly different ($P<0.05$). SED is based on within-volunteer spread. Sleep latency and sleep disturbance the reported SED is actually SE of the ratio between diets due to using log transformed values. Sleep quality score range: 1-9, 1 = best, 9 = worst. Actual sleep duration based on difference between sleep time and wake time subtracting away sleep latency and participant estimated duration of night-time awakenings. Sleep disturbances refers to the number of times an individual awoke during the night.

EL: evening loaded, ML: morning loaded, SED: standard error of the difference; averaged over pairs when comparing more than two treatments

Supplemental Table 4: Gastric emptying results, related to Figure 4

	Baseline	Washout	ML	EL	SED	P
T_{asc} (h:mm)	3:57 [ab]	3:43 [a]	4:38 [b]	2:50[c]	0:17	<0.001
T_{half} (h:mm)	6:08[a]	5:56 [a]	6:25[a]	4:07[b]	0:20	<0.001
T_{lag} (h:mm)	4:37 [a]	4:29 [a]	4:23[a]	2:57[b]	0:14	<0.001
T_{lat} (h:mm)	2:11[a]	2:12 [a]	1:46[b]	1:17[c]	0:10	<0.001

Gastric emptying measured using the ^{13C} octanoic acid breath test.

Analyzed for diet effect by ANOVA, means in the same row not sharing a superscript are significantly different (P<0.05). SED is based on within-volunteer spread.

The data was fitted to the $mk\beta$ model presented in Ghoos et al (Ghoos et al., 1993): $y = m(1 - e^{-kt})^{\beta}$, where y is the cumulative percentage dose of $^{13}CO_2$ recovered at time-point t (mins), and $m, k,$ and β are constants with m being the total cumulative $^{13}CO_2$ recovery when time is infinite. All modelling (curve-fitting) was done in R (version 4.0.5) using the nonlinear least square's function.

EL: evening loaded, ML: morning loaded, SED: standard error of the difference; averaged over pairs when comparing more than two treatments, T_{asc}: the length of time during which $^{13}CO_2$ excretion in the breath is rapid i.e. when the cumulative curve is ascending, T_{half}: timepoint at which 50% of the total excretion of $^{13}CO_2$ in the breath has been recovered, T_{lag}: timepoint at which $^{13}CO_2$ excretion rate in the breath is at its maximum, T_{lat}: initial delay or latency of $^{13}CO_2$ excretion in the breath

Supplemental Table 5. Glucose parameters calculated from continous glucose monitors, related to Figure 5

	Baseline	Washout	ML	EL	SED	P_{diet}	$P_{wt.loss}$	$P_{diet*wt.loss}$
MAGE (mmol/L)	3.03	3.02	2.56	2.32	0.19	<0.001	<0.001	0.389
Mean (mmol/L)								
Whole day	6.13	5.96	5.62	5.67	0.10	<0.001	<0.001	0.598
Breakfast	7.06	6.80	6.34	6.10	0.15	<0.001	<0.001	0.213
Lunch	6.92	6.86	6.41	6.31	0.18	0.001	<0.001	0.860
Dinner	6.99	9.72	6.07	6.26	0.16	<0.001	<0.001	0.410
SD (mmol/L)								
Whole day	0.96	0.96	0.72	0.71	0.06	<0.001	<0.001	0.717
Breakfast	0.66	0.69	0.64	0.60	0.06	0.420	0.324	0.396
Lunch	0.76	0.81	0.59	0.66	0.08	0.018	0.003	0.586
Dinner	0.74	0.75	0.53	0.52	0.07	0.003	<0.001	0.408
AUC (mmol • min/L)								
Whole day	8793	8559	8053	8133	140	<0.001	<0.001	0.589
Breakfast	815	785	732	704	18	<0.001	<0.001	0.224
Lunch	799	792	740	729	21	0.001	<0.001	0.859
Dinner	807	775	700	721	18	<0.001	<0.001	0.423
iAUC (mmol • min/L)								
Whole day	1079	849	684	581	106	0.005	<0.001	0.485
Breakfast	120	127	90	71	11	<0.001	<0.001	0.050
Lunch	152	153	103	108	17	0.003	<0.001	0.824
Dinner	125	135	86	101	17	0.018	0.007	0.242
Minimum (mmol/L)								
Whole day	4.55	4.51	4.47	4.48	0.15	0.981	0.683	0.994
Breakfast	5.93	5.63	5.41	5.24	0.12	<0.001	<0.001	0.052
Lunch	5.52	5.43	5.43	5.27	0.13	0.429	0.346	0.391
Dinner	5.71	5.45	5.23	5.32	0.11	0.001	<0.001	0.103
Maximum (mmol/L)								
Whole day	8.70	8.66	7.81	7.86	0.23	<0.001	<0.001	0.814
Breakfast	7.97	7.77	7.32	6.99	0.21	<0.001	<0.001	0.286
Lunch	7.87	7.90	7.22	7.25	0.25	0.003	<0.001	0.952
Dinner	7.96	7.76	6.86	6.99	0.23	<0.001	<0.001	0.824

Glucose indices measured with continuous glucose monitors during a 2 to 3-day period at the end of each dietary phase. Whole day represents 24-hour measures between 00:00 until 00:00 the

following day. Breakfast, lunch and dinner measurements taken from the 2-hour period following the timing of meals recorded by participants. Analyzed using linear mixed models.

EL: evening loaded, MAGE: mean amplitude of glycaemic excursions, ML: morning loaded, SED: standard error of the difference; averaged over pairs when comparing more than two treatments

Supplemental Table 6. Fasting and 2-hour plasma glucose and lipids, as described in STAR methods.

	Time (h)	Baseline	Washout	ML	EL	SED_{diet}	$SED_{time*diet}$	$P_{diet}{}^{2}$	$P_{time*diet}{}^{2}$
Cholesterol (mmol/L)	0	5.50^{a}	4.86^{b}	4.92^{bc}	4.94^{bc}				
	2	5.63^{a}	5.00^{c}	5.00^{bc}	4.84^{bc}				
	ALL	5.56^{a}	4.93^{b}	4.96^{b}	4.89^{b}	0.14	0.15	<0.001	0.043
HDL-Cholesterol (mmol/L)	0	1.28	1.17	1.27	1.22				
	2	1.24	1.17	1.26	1.22				
	ALL	1.26^{a}	1.17^{b}	1.26^{a}	1.22^{ab}	0.36	0.04	0.037	0.415
LDL-Cholesterol (mmol/L)	0	3.26	2.84	2.97	2.90				
	2	3.23	2.83	2.94	2.94				
	ALL	3.24^{a}	2.84^{b}	2.95^{b}	2.92^{b}	0.11	0.11	0.002	0.390
TC-HDL Cholesterol Ratio	0	4.71^{a}	4.35^{bd}	4.04^{c}	4.23^{bcd}				
	2	4.93^{a}	4.54^{ab}	4.18^{d}	4.13^{cd}				
	ALL	4.82^{a}	4.45^{ab}	4.11^{b}	4.18^{b}	0.21	0.22	0.002	0.003
LDL-HDL Cholesterol Ratio	0	2.82	2.54	2.45	2.48				
	2	2.84	2.57	2.45	2.53				
	ALL	2.83	2.55	2.45	2.51	0.14	0.15	0.081	0.583
NEFA (mmol/L)	0	0.59	0.60	0.75	0.67				
	2	0.21	0.17	0.25	0.25				
	ALL	0.40^{a}	0.38^{a}	0.50^{b}	0.46^{b}	0.03	0.04	<0.001	0.102
Triglycerides (mmol/L)	0	1.77^{a}	1.38^{c}	0.97^{d}	0.99^{d}				
	2	2.35^{b}	1.96^{a}	1.24^{c}	1.11^{d}				
	ALL	2.06^{a}	1.67^{b}	1.11^{c}	1.05^{c}	0.10	0.11	<0.001	<0.001
Glucose (mmol/L)	0	5.70	5.30	5.27	5.30				
	2	5.49	5.17	4.73	4.55				
	ALL	5.60^{a}	5.24^{b}	5.00^{bc}	4.92^{c}	0.12	0.17	<0.001	0.132

Blood metabolites were log transformed prior to being analyzed by hierarchical ANOVA. Means for each metabolite for 0 h and 2 h measures not sharing a superscript are significantly different (P<0.05). Means within each row specified *ALL* not sharing a superscript are significantly different (P<0.05).

EL: evening loaded, ML: morning loaded, NEFA: non-esterified fatty acids, SED: standard error of the difference; averaged over pairs when comparing more than two treatments, TC: total cholesterol
