# Supplementary material for: Timing of daily calorie loading affects appetite and hunger responses without changes in energy metabolism in healthy subjects with obesity
Source: Cell Metab. 2022 Oct 4;34(10):1472–1485.e6. doi: 10.1016/j.cmet.2022.08.001 (PMC9605877; doi:10.1016/j.cmet.2022.08.001)
Supplement: Document S3. Article plus supplemental information [file mmc3.pdf]

Cell Metabolism
Clinical and Translational Report

Timing of daily calorie loading affects appetite and hunger responses without changes in energy metabolism in healthy subjects with obesity

Graphical abstract

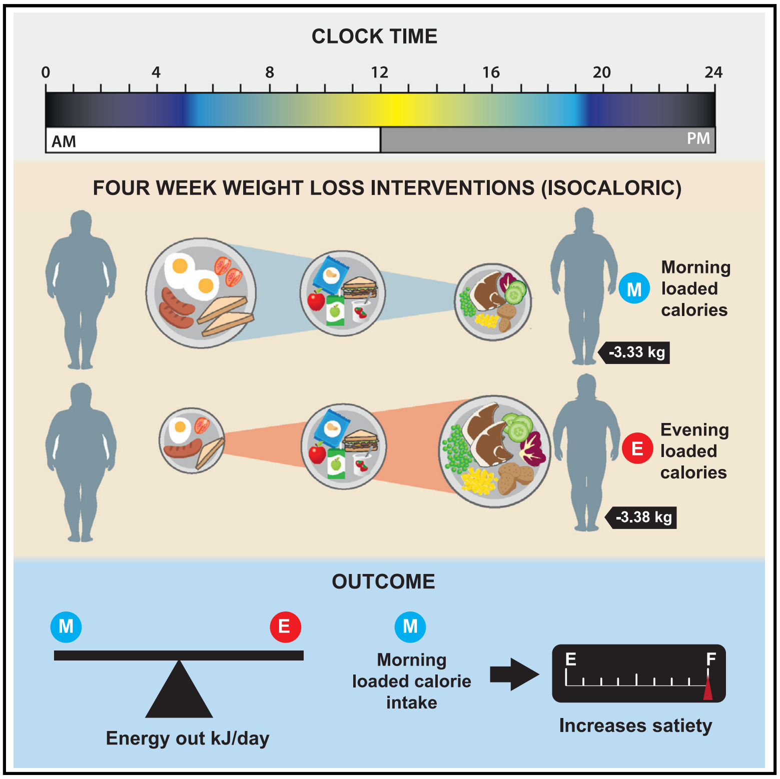

Authors

Leonie C. Ruddick-Collins, Peter J. Morgan, Claire L. Fyfe, ..., Klaas R. Westerterp, Jonathan D. Johnston, Alexandra M. Johnstone

Correspondence

alex.johnstone@abdn.ac.uk

In brief

Ruddick-Collins et al. studied the effects of daily calorie distribution on energy balance, metabolism, and appetite. Weight loss and energy expenditure were similar regardless of calorie distribution. Morning loaded calorie intake resulted in greater appetite suppression, which may lead to greater weight loss under *ad libitum* eating conditions.

Highlights

- Morning loaded (ML) versus evening loaded (EL) energy intake diets were compared
- Both diets produced similar weight loss, with no differences in energy metabolism
- ML energy intake resulted in lower daily subjective appetite and hunger
- Behavioral changes (reduced intake) likely explain weight loss benefits of ML diets

Ruddick-Collins et al., 2022, Cell Metabolism *34*, 1472–1485
October 4, 2022 © 2022 The Author(s). Published by Elsevier Inc.
https://doi.org/10.1016/j.cmet.2022.08.001

CellPress
OPEN ACCESS

Cell Metabolism

Clinical and Translational Report

Timing of daily calorie loading affects appetite and hunger responses without changes in energy metabolism in healthy subjects with obesity

Leonie C. Ruddick-Collins,[1] Peter J. Morgan,[1] Claire L. Fyfe,[1] Joao A.N. Filipe,[3] Graham W. Horgan,[3] Klaas R. Westerterp,[4] Jonathan D. Johnston,[2] and Alexandra M. Johnstone[1,5,*]

[1]The Rowett Institute, University of Aberdeen, Foresterhill Road, Aberdeen AB25 2ZD, UK
[2]Section of Chronobiology, Faculty of Health and Medical Sciences, University of Surrey, Guildford GU2 7XH, UK
[3]Biomathematics & Statistics Scotland, Foresterhill Road, Aberdeen AB25 2ZD, UK
[4]NUTRIM, Maastricht University Medical Centre, Maastricht 6229 HX, the Netherlands
[5]Lead contact
*Correspondence: alex.johnstone@abdn.ac.uk
https://doi.org/10.1016/j.cmet.2022.08.001

SUMMARY

Morning loaded calorie intake in humans has been advocated as a dietary strategy to improve weight loss. This is also supported by animal studies suggesting time of eating can prevent weight gain. However, the underlying mechanisms through which timing of eating could promote weight loss in humans are unclear. In a randomized crossover trial (NCT03305237), 30 subjects with obesity/overweight underwent two 4-week calorie-restricted but isoenergetic weight loss diets, with morning loaded or evening loaded calories (45%:35%:20% versus 20%:35%:45% calories at breakfast, lunch, and dinner, respectively). We demonstrate no differences in total daily energy expenditure or resting metabolic rate related to the timing of calorie distribution, and no difference in weight loss. Participants consuming the morning loaded diet reported significantly lower hunger. Thus, morning loaded intake (big breakfast) may assist with compliance to weight loss regime through a greater suppression of appetite.

INTRODUCTION

Current dietary advice for weight management is broadly based on the assumption that a "calorie is a calorie" no matter when those calories are consumed across the day. However, recent dietary intervention studies in humans (Garaulet et al., 2013; Jakubowicz et al., 2013) have challenged this assumption, suggesting that the time of day when a large meal is consumed may influence weight loss effectiveness. These studies imply that calories ingested at different times of the day have different effects on energy utilization, leading to differential weight loss, even when consumed at iso-caloric amounts. In the study by Jakubowicz et al. (2013), a group of 93 overweight women were fed iso-caloric diets over 12 weeks, with morning loaded and evening loaded calorie intakes run in a parallel design. Those who consumed most of the calories in the morning relative to the evening lost an additional 5.1 kg weight overall. Similarly, Garaulet et al. (2013) reported that "late" eaters lost less weight than "early" eaters, over a 20-week protocol, resulting in a 2.2 kg difference in body mass loss, even when reportedly consuming similar calories. These studies imply that calorie utilization varies across the day, with calories consumed in the morning being less efficiently utilized than calories consumed in the evening, resulting in greater energy expenditure (EE) relative to intake and thus enabling more effective weight loss. With recent studies linking energy regulation to the circadian clock at the behavioral, physiological, and molecular levels (Garaulet and Gómez-Abellán, 2014; Jakubowicz et al., 2013), there is considerable interest in time-of-day-dependent changes in metabolism. Indeed, when experimental animals are fed a high-fat diet during daylight hours (the "wrong" time for nocturnal rodents), they become obese despite consuming the same amount of energy relative to those fed during the dark (the "correct" feeding time for nocturnal rodents) (Arble et al., 2009). Other labs have reported comparable findings for mice (Fonken et al., 2010) and Zucker rats (Sensi and Capani, 1987) with similar conclusions. These rodent data support the notion that the timing of calorie intake may offer benefits to dietary approaches used for the management of obesity in humans. However, the potential mechanisms involved in any differential calorie utilization across the day remain undetermined.

Since timing and distribution of food intake is a modifiable lifestyle behavior, there is interest in understanding if timing of eating and distribution of food intake influences the success of weight-loss diets in humans. A recent randomized control trial (RCT) (Cienfuegos et al., 2020) on time-restricted feeding (TRF) as a popular form of intermittent fasting assessed weight loss

 © 2022 The Author(s). Published by Elsevier Inc.
This is an open access article under the CC BY license (http://creativecommons.org/licenses/by/4.0/).

effectiveness and reported that both 4 h TRF (eating between 3 and 7 p.m.) and 6 h TRF (eating only between 1 and 7 p.m.) can produce similar reductions in body weight (~3%), linked to calorie reduction, relative to a control group. Other human studies (Carlson et al., 2007; Gill and Panda, 2015; Moro et al., 2016; Stote et al., 2007; Tinsley et al., 2017) using 4–10 h TRF have reported that the results of TRF may be linked to the time of day of feeding. For example, a calorie intake window during the middle of the day reportedly reduced body weight or body fat (Gill and Panda, 2015; Moro et al., 2016); conversely, restricting food intake to the late afternoon or evening produced mostly null results (Carlson et al., 2007; Stote et al., 2007; Tinsley et al., 2017). Interestingly, Sutton et al. (2018) report that early TRF (morning eating) influences appetite control by lowering the desire to eat in the evening, to facilitate weight loss. Studies in rodents (using intermittent fasting windows of 3–10 h) also report that TRF reduces body weight and increases EE relative to grazing on food throughout the day (Belkacemi et al., 2012; Hatori et al., 2012; Olsen et al., 2017; Sherman et al., 2012; Woodie et al., 2018; Wu et al., 2011; Zarrinpar et al., 2014).

The circadian system may explain why the effects of TRF appear to depend on the time of day, since food intake is known to be a "zeitgeber" and has been shown to entrain peripheral clocks (Flanagan et al., 2021). To our knowledge, studies applying objective measures in humans to examine the role of both timing and calorie distribution on weight loss have not been conducted. One way to examine this is a highly controlled RCT in humans in which all food and beverages are provided by the lab and EE is measured objectively, using the doubly labeled water technique (DLW) (Westerterp, 2017). There are three potential mechanisms that could account for differential energy utilization leading to differential weight: (1) behavioral adaption, such as altered appetite or physical activity response to a calorie load; (2) alteration in energy metabolism at other times of the day in response to meal timing; or (3) the influence of normal biological circadian/diurnal rhythms on energy metabolism at different times of the day. Energy metabolism can be considered the amount of energy (kJ or kcal) that an individual uses in a given time, and this can be divided into three components: (1) basal metabolic rate (BMR), the minimum calories the body utilizes at rest to maintain normal body functions; (2) thermic effect of food (TEF), which is the energy cost of absorbing, processing, and storing nutrients after food ingestion; and (3) energy expended in physical activity. Physical activity is any body movement that works the skeletal muscles and requires more energy than resting (Ruddick-Collins et al., 2020). By measuring all components of EE during a controlled dietary study, changes in energy balance leading to weight loss can be accounted for.

This is the first within-subject RCT to compare the weight-loss efficacy of meal timing and calorie distribution and assess all components of EE that could impact energy balance and weight loss in healthy but obese persons. We measured and monitored changes in EE, resting metabolic rate (RMR by indirect calorimetry), post-prandial thermogenesis (TEF by indirect calorimetry), free-living total EE using DLW, and physical activity using accelerometry. The objective of this study was to measure these components of energy metabolism while subjects consumed controlled diets, with the largest meal of the day at breakfast (with a smaller evening meal) in comparison to an evening loaded meal (with a smaller breakfast meal). We hypothesized that the calorie distribution of morning loaded energy intake (EI) as a big breakfast and small dinner would produce a greater weight loss linked to either behavioral or circadian influence on energy metabolism, even when fed iso-energetic amounts.

RESULTS AND DISCUSSION

We performed a 4-week randomized crossover isocaloric and eucaloric controlled feeding trial, comparing morning loaded (ML; 45%:35%:20% calories at breakfast:lunch:dinner) versus evening loaded (EL; 20%:35%:45% calories at breakfast:lunch:dinner) calorie intake (Figure 1A). Individual calorie intake was fixed, referenced to each individual's measured RMR to assess the effect of meal timing under isoenergetic intake on weight loss and EE. All food and beverages were provided, making this the most rigorously controlled study to assess timing of eating in humans to date, with the aim of accounting for all aspects of energy balance. In brief, diets were designed to provide the same nutrient composition (30%:35%:35% as protein:carbohydrate:fat), fed to 1.0× RMR. A controlled baseline diet (B) was provided to standardize diets before intervention, fed to maintain energy balance (1.5× RMR), provided as 33% of calorie intake fed at breakfast, lunch, and dinner. The same washout (W) diet was provided in the 7-day period between weight loss interventions. Seven days were applied as a pragmatic decision to maintain energy balance, but also, in consideration of the study duration, to maintain compliance with volunteers eating the diets provided. The primary end point was energy balance measured by body weight, which we considered to be driven by altered EE since the study provided iso-caloric diets. Secondary end points were subjective appetite control, glycemic control (continuous glucose monitoring and plasma profile), and body composition. Weight loss (denoted by Δ) was determined as the within-diet change in body weight (from the first to the final day of the 4-week weight-loss diets). All other differences between meal timing schedules were assessed by comparing outcomes measured within the last week of each diet. The average times that the meals were consumed for the ML and EL meals are shown in Figure S1.

Participants

As shown in Figure 1B, 37 individuals attended the clinic for screening after responding to a social media advertisement. Adults were recruited to be overweight/obese (BMI 27–42 kg/m^2) but otherwise healthy. Of these participants, 6 were excluded because they did not meet one or more of the inclusion criteria and/or declined to participate further. Inclusion and exclusion criteria are detailed in the STAR Methods section. A total of 31 participants were randomized into the study with 1 participant dropping out before completion of both diets due to work commitments. Thirty participants (mean ± SEM; age, 50.9 ± 2.1 years; weight, 95.4 ± 3.1 kg; BMI, 32.5 ± 0.7 kg/m^2) completed the study (16 male, 14 female), with 14 randomized to the ML diet first and 16 to the EL diet first. Further pre-intervention characteristics are presented in Table S1 and baseline measurements are in Tables 1 and 2. There were no serious adverse events related to the protocol.

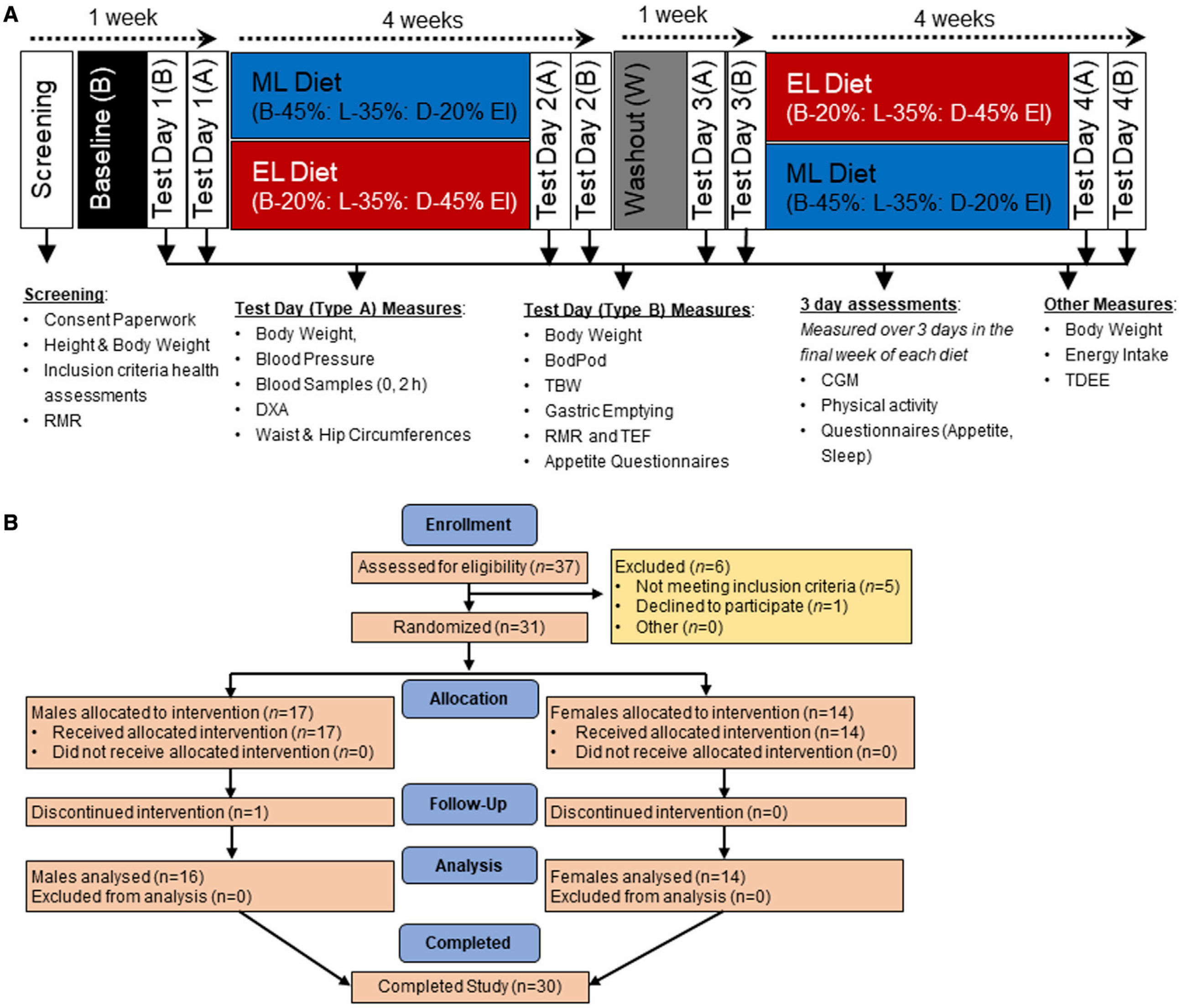

Figure 1. Study design and CONSORT flow diagram

(A) Study design. Prior to baseline, participants completed comprehensive in-lab screening to determine their eligibility. Eligible participants were randomly assigned to the order in which they received the morning loaded (ML) and evening loaded (EL) weight-loss diets. The baseline (B) and washout (W) diets were provided as energy intake (EI) equally distributed between breakfast, lunch, and dinner (33% of EI at all meals). All meals were provided from the start of baseline to the end of the study. EI during B and W was 1.5× measured RMR. EI during ML and EL weight-loss diets was 1.0× RMR. EI was measured over the entire study duration based on food provided and daily food records. Body weight was assessed 3 times per week when participants attended the institute to collect their meals. Total daily energy expenditure (TDEE) was measured for the entire 4-week duration of each weight-loss diet using doubly labeled water (DLW). All other measures were assessed in the final week of each dietary phase.

(B) Consort diagram describing the number of patients throughout the process of enrollment through to completion.

RMR, resting metabolic rate; DXA, dual-energy X-ray absorptiometry; TBW, total body water; TDEE, total daily energy expenditure; TEF, thermic effect of food; DLW, doubly labeled water.

Diet provision

Participants reported any foods they consumed outside the prescribed diet and all leftovers were weighed. As per protocol, there were no significant differences in total calorie intake between the ML (1,736 kcal/day) and EL (1,749 kcal/day) diets, with both significantly lower than B (2,658 kcal/day) and W (2,474 kcal/day, standard error of the difference [SED] 72.7 kcal/day, $p < 0.05$) diets. Additionally, there were no differences in macronutrient intake between the ML (protein:carbohydrates:fat, 29.7%:34.2%:34.7%) and EL diets (29.8%:34.0%:34.8%) (Table S2). Leftovers were significantly higher at breakfast and lunch while on the ML versus EL diet (mean ± SEM; breakfast, 85.5 ± 6.1 kcal/day versus 24.5 ± 2.4 kcal/day, $p < 0.001$; lunch, 73.3 ± 6.5 versus 54.2 ± 7.7 kcal/day, $p = 0.03$) and modestly lower at dinner on the ML versus EL diet (149.4 ± 19.3 versus 173.5 ± 18.3 kcal/day, $p = 0.18$). Despite this, calorie intake across the day remained almost perfectly inverse with an overall energy distribution of 38.4%:35.4%:26.2% (breakfast:lunch:dinner) on the ML diet and 26.3%:35.3%:38.4%

Table 1. Anthropometric data measured at the end of each diet

	Baseline	Washout	ML	EL	SED	p
Body weight (kg)	95.4*	92.0**	90.4***	90.3***	0.51	<0.001
BMI (kg/m^2)	32.5*	31.4**	30.8***	30.8***	0.16	<0.001
Percent body fat	41.54*	39.24**	38.49**	38.60**	2.14	<0.001
Weight loss (kg)	–	–	−3.33	−3.38	0.24	0.848
Waist circumference (cm)	107.0*	102.7**	101.4***	100.8***	0.57	<0.001
Hip circumference (cm)	112.9*	110.5**	109.6**	110.3**	0.47	<0.001
WHR	0.95*	0.93**	0.92**,***	0.91***	0.01	<0.001

Data are expressed as means, n = 30. Analyzed for diet effect by hierarchical ANOVA (linear mixed models); means in the same row not sharing a superscript are significantly different (p < 0.05). SED is based on within-volunteer spread. Weight loss calculated as the weight change from the end of the preceding maintenance diet (B or W) to the end of each weight loss diet. EL, evening loaded; ML, morning loaded; SED, standard error of the difference, averaged over pairs when comparing more than two treatments; WHR, waist-hip ratio.

(breakfast:lunch:dinner) on the EL diet. On average, participants' recorded mealtimes on the ML versus EL weight-loss diets were similar at breakfast (ML, 07:43 h; EL, 07:52 h; SED 00:07 h, NS) and lunch (ML, 13:10 h; EL, 12:58 h; SED, 00:07 h NS), with dinner tending to be slightly (~20 min) earlier on the EL diet (ML, 18:51 h; EL, 18:32 h; SED 00:07 h, $p < 0.05$). This resulted in a modestly longer eating window across the day in the ML compared to EL diet (ML, 11:05 h; EL, 10:39 h; SED 00:09 h, $p < 0.05$) (full data in Table S3).

ML calorie intake does not result in greater weight loss compared to EL calorie intake

We observed that two isocaloric diets with the same composition, differing in energy distribution, resulted in significant weight reduction at the end of each dietary intervention period (Table 1). Weight loss (Figure 2A) showed a near-identical downward trend, with no difference in weight loss at the end of the 4 weeks, between the ML and EL diets (ML, Δ−3.33 kg; EL, Δ−3.38 kg; SED 0.24 kg, p = 0.848). In contrast, previous work (Garaulet et al., 2013; Jakubowicz et al., 2013), conducted as a parallel group design, reported significantly greater weight loss linked to earlier calorie intake. However, similar results to our own have also been previously reported in a parallel arm study by Versteeg et al. (2018), who found no significant differences in weight loss between ML (50% EI at breakfast) and EL (50% EI at dinner) feeding. Previous work was completed in females (Jakubowicz et al., 2013), males (Versteeg et al., 2018), or both genders (Garaulet et al., 2013); thus, gender effects appear not to account for any differences between the studies. In our current within-subject comparison we included both genders; however, our study was not powered to examine gender differences. The differences in the results between studies may be related to study duration with the studies by Jakubowicz et al. (2013) and Garaulet et al. (2013), which were substantially longer in duration (12 weeks and 20 weeks, respectively) compared to our own and the Versteeg et al. (2018) trial (both 4 weeks). Notably, although statistically significant differences in weight loss were evident by week 4 in the study by Jakubowicz, in the Garaulet study, late eaters only began to display a slower rate of weight loss starting after the 5th week of treatment. It is therefore possible that we may have found more striking effects in terms of weight loss over a longer duration. It is also feasible that the study design and rigidity of measuring calorie intake may have contributed to the different results. Garaulet et al. (2013) retrospectively grouped participants into "early eaters" and "late eaters," according to the timing of the main meal (lunch time before and after 1500 h, respectively); food intake and physical activity were subjectively recorded (food diary and international physical activity questionnaire, IPAQ). Jakubowicz et al. (2013) also applied a meal plan methodology (provision of dietary advice and food choices for self-implementation) and assessed intake via a participant recorded, weekly 3-day record. In comparison, our results provide rigorous and objective assessment of both EI and expenditure, with all meals provided and participants attending the unit 3 days per week for the entire duration of the study for regular body mass measurement, enabling us to regularly track weight-loss progress and monitor compliance. Our study also applied the gold standard 4-compartment method (Fuller et al., 1992) to measure body composition, which showed that compositional weight changes, in addition to weight loss, were also similar and independent of diet calorie distribution (Figure S2).

An important consideration is the window and duration over which feeding occurs across the day. In our own study, the eating window was 11:05 h and 10:39 h in the ML versus EL diets, respectively, with meals consumed relatively early in the day. Gill and Panda (2015) were the first to show that 16 weeks of TRF in overweight participants induced a modest weight loss (~3% body mass) when the eating window was reduced from >14 h to 10 h per day. However, with only small subject numbers (n = 8) and no control group, wider interpretation of these results is limited. The precise mechanism(s) of how TRF improves health outcomes in the absence of enforced energy restriction is unknown.

ML calorie intake lowers appetite and hunger compared to EL calorie intake

The effect of meal timing on appetite control for weight loss is poorly understood (Beaulieu et al., 2020), with studies often lacking temporal measures of appetite. In this study, we assessed changes in subjective appetite using visual analog scales (VASs), measured hourly from the time of waking until bedtime for 3 consecutive days in a free-living context, followed by an in-lab test day consisting of 30 min continuous measures. The ML diet resulted in significantly lower average daily hunger, desire to eat, prospective consumption, thirst, and composite

Table 2. Metabolic health parameters measured at the end of each diet

	Baseline	Washout	ML	EL	SED	p
Blood pressure						
SBP (mmHg)	127*	120**	119**	119**	1.82	<0.001
DBP (mmHg)	81*	76**	76**	75**	1.14	<0.001
Pulse (beats/min)	69*	63**	62**	64**	1.11	<0.001
Glucoregulatory factors						
Fasting glucose (mmol/L)	5.70*	5.30**	5.27**	5.30**	0.09	<0.001
Fasting insulin	7.61*	5.97**	5.37**	5.70**	0.34	<0.001
HOMA-IR	1.72*	1.34**	1.18**	1.22**	0.10	<0.001
HbA1c mmol/mol[a]	35.3*	35.5*	33.0**	33.2**	0.65	<0.001
Fasting plasma lipids						
Cholesterol (mmol/L)	5.50*	4.86**	4.92**	4.94**	0.15	<0.001
HDL-cholesterol (mmol/L)	1.28*	1.17**	1.27*	1.22*,**	0.04	<0.001
LDL-cholesterol (mmol/L)	3.26*	2.84**	2.97**	2.90**	0.01	<0.001
NEFAs (mmol/L)	0.59*	0.60*,**	0.75***	0.67**	0.04	0.05
Triglycerides (mmol/L)	1.77*	1.38**	0.97***	0.99***	0.09	<0.001
Energy expenditure						
TDEE (DLW)	–	–	2,871	2,846	100.7	0.184
RMR (kcal)	1,821*,**	1,729**,***	1,675***	1,690***	36	<0.001
Breakfast TEF (kcal)	131*,**	116*,***	147**	98***	8	<0.001
Breakfast TEF (% of EI)	15.4*	14.0*	19.0**	27.6***	1.5	<0.001
Sleep and activity by accelerometry						
Average daily steps	6,611	6,966	6,992	7,046	449	0.762
Activity EE (kcals/day)	1,740	1,599	1,585	1,583	97	0.312
Sleep duration (min)	426	428	428	422	8.31	0.888

Data are expressed as means, n = 30. Means not sharing a superscript are significantly different ($p < 0.05$). DBP, diastolic blood pressure; EL, evening loaded; HOMA-IR, homeostasis model assessment of insulin resistance; ML, morning loaded; RMR, resting metabolic rate; SBP, systolic blood pressure; SED, standard error of the difference, averaged over pairs when comparing more than two treatments; TDEE, total daily energy expenditure, measured by DLW; TEF, thermic effect of food.
[a]HbA1c estimated from CGM data

appetite score (calculated as (hunger + desire to eat + food quantity + (100 − fullness))/4; Anderson et al., 2002) compared to the EL diet (Figure 3A). The temporal appetite curves plotted as hourly averages over 3 consecutive days (Figure 3B) and every 30 min following in-lab test breakfasts (Figure 3C) reinforce these findings. This is consistent with the study of Jakubowicz et al. (2013), which also reported significantly lower overall hunger scores and significantly higher mean satiety scores measured across a full day in response to 3 meals in the ML relative to the EL group. Under *ad libitum* feeding conditions, it therefore seems possible that greater suppression of appetite from the use of ML diets may result in reduced calorie intake at lunch and dinner, thereby contributing to enhanced weight loss with ML diets.

Several human studies, including those using forced desynchrony protocols, provide evidence for an endogenous circadian rhythm in appetite, with greater evening hunger independent of wake time and calorie intake (Sargent et al., 2016; Scheer et al., 2013; Wehrens et al., 2017). Despite this greater evening hunger, it has been suggested that the satiety value of food may be greater earlier relative to later in the day (de Castro, 1987). Under *ad libitum* conditions, particularly in the UK, calorie intake increases as the day progresses as a result of larger meals sizes and lower time intervals between meals (Almoosawi et al., 2012; de Castro, 2004).

Recent work by Beaulieu et al. (2020) assessed appetite and food reward after an identical ‘‘early’’ and ‘‘late’’ meal in both early and late chronotypes and reported lower appetite during the morning test session and greater perceived ability to promote feelings of fullness after the test meal. Additionally, they reported significantly greater perceived meal fullness in morning versus evening chronotypes. In the evening, participants had greater liking and desire for high-fat foods, irrespective of chronotype, albeit there was an overall greater wanting for high-fat food in evening chronotypes. In the current study, based on the same morningness-eveningness questionnaire (MEQ) (Horne and Östberg, 1976), our participants were classified as neither morning nor evening types (MEQ type, 3.7 ± 0.2; range, 2–5 [1 = definitely evening to 5 = definitely morning]). Although we report statistically significant changes in sleep latency and disturbance (Table S3) associated with weight loss, as changes from baseline, these are not clinically significant and were not different between EL and ML diets. We may have detected different patterns of ‘‘morning-responsive’’ appetite suppression

CellPress
OPEN ACCESS

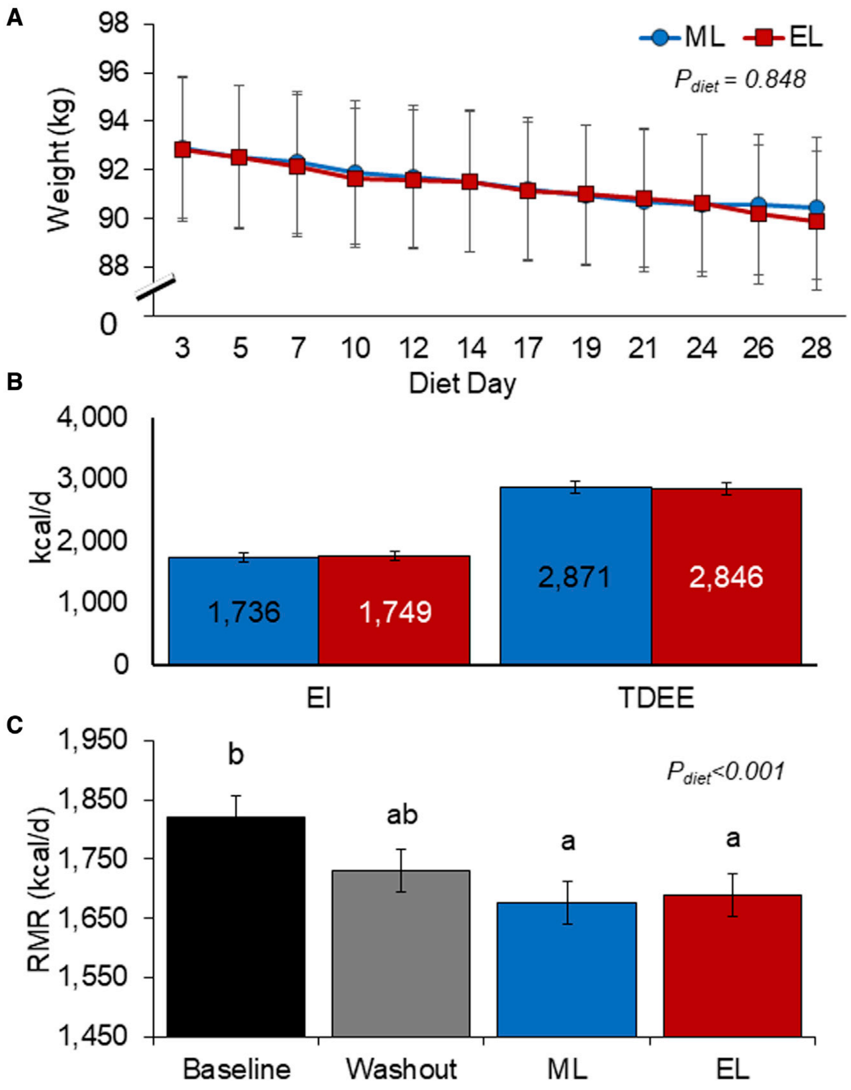

Figure 2. ML and EL weight-loss diets resulted in similar weight loss and comparable EE when EI was controlled

(A) Weight loss over the duration of the 4-week weight-loss diets.

(B) EI and TDEE averaged over the 4 weeks of the ML and EL diets.

(C) RMR measured at the end of each dietary phase. Means were estimated using hierarchical ANOVA (linear mixed models). Values expressed as mean ± SEM. Bars with different letters are significantly different ($p < 0.05$). EI, energy intake; EL, evening loaded; ML, morning loaded; RMR, resting metabolic rate; TDEE, total daily energy expenditure.

if we had recruited participants based on chronotype. There is further scope to examine the interaction of chronotype on appetite control for weight loss. Our data support the notion that meal timing should be considered in the assessment and interpretation of appetite and food reward outcomes, along with chronotype, to support eating behaviors to promote healthy weight.

Circadian and/or diurnal variation is also evident in fasting and postprandial secretion of many appetite hormones (Ruddick-Collins et al., 2020). For example, postprandial ghrelin (traditionally considered the "hunger hormone") is suppressed to a greater extent in the morning in response to a breakfast meal, as compared to the same meal provided in the evening (Qian et al., 2019). However, how daily energy distribution influences appetite hormones throughout the day is not entirely clear. Although our study demonstrates no differences in fasting PYY, ghrelin, GIP, and GLP-1 between the ML and EL diets (Figure 4A), the 2 h postprandial ghrelin response was significantly lower, with PYY, GIP, and GLP-1 (satiety-enhancing GI hormones) significantly higher after the ML breakfast compared to the EL breakfast. While these data largely reflect the difference in meal size, they correspond with the subjective appetite responses of prolonged appetite suppression associated with ML feeding.

These gut hormones play a critical role in modulating appetite and the rate of gastric emptying (which slows in the evening). We report greater appetite suppression with the ML diet, which is likely in part due to the extended duration of gastric emptying of the larger meal size and calorie load (Figure 4B). We assessed gastric emptying using stable isotope ^{13}C octanoic acid breath test, with breath samples collected for 6 h after the ML and EL breakfast test meal. Following the smaller breakfast meal with the EL diet, half time (T_{half}; the time taken for half the meal to empty from the stomach) was reached within 4:23 h, which was significantly quicker than the larger breakfast meal on the ML diet, which exceeded the 6 h measurement period, with an estimated T_{half} of 6:25 h (SED 0:20 h:mm, $p < 0.001$) (Table S4). Similarly, compared to breakfast on the EL diet, the ML breakfast resulted in a significantly longer lag time (T_{lag}; the time point at which the rate of excretion reaches its maximum) (4:23 versus 2:57 h:mm, SED 0:14 h:mm, $p < 0.001$), latency (T_{lat}; the initial delay in gastric emptying) (1:46 versus 1:17 h:mm, SED 0:10 h:mm, $p < 0.001$), and ascending time (T_{asc}; duration for which excretion from the gut is ascending) (4:38 versus 2:50 h:mm, SED 0:17 h:mm, $p < 0.001$). Overall, these parameters indicate a longer time for the meal to begin emptying from the gut and extended time for the rate of gastric emptying to increase to its maximum. Diurnal variation in gastric emptying rates has long been established, with significantly longer T_{half} for solid meals reported in the evening compared to the morning (Goo et al., 1987). Thus, a small breakfast more rapidly empties from the stomach, leaving the consumer hungry again sooner. A larger meal in the morning could help increase the content remaining in the gut and duration of emptying, therefore reducing the likelihood of overeating later in the day. Our data are consistent with other reports that have used similar isotopic technique to assess the role of gastric emptying in "early" and "late" mealtime during TRF (Hutchison et al., 2019). They also demonstrate that gastric

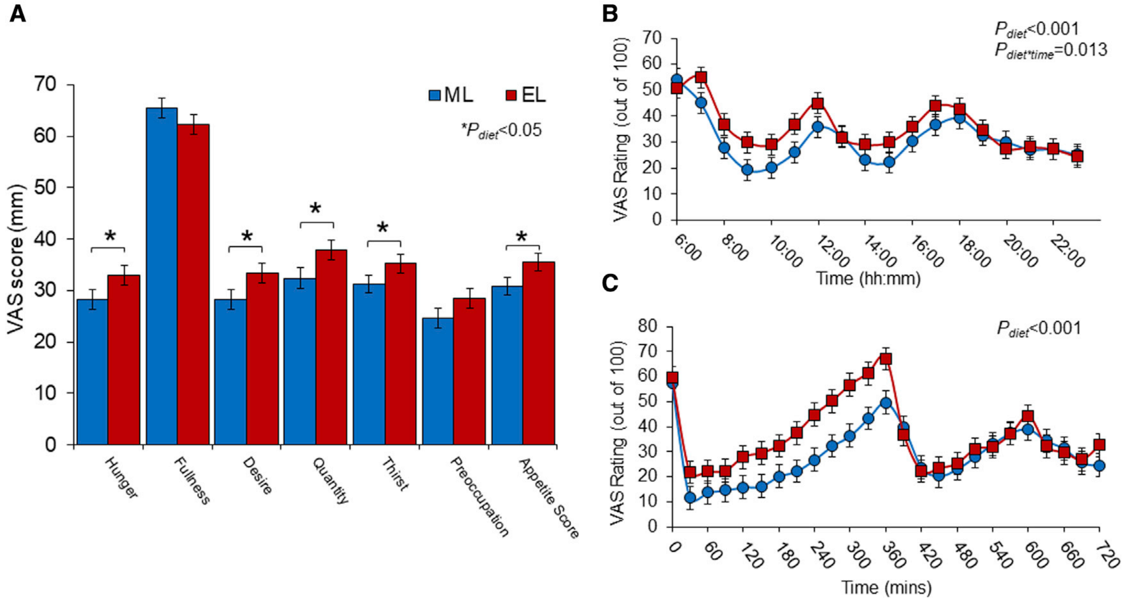

Figure 3. ML weight-loss diet resulted in significantly lower hunger and appetite compared to EL weight-loss diet

(A) Average appetite scores from hourly free-living appetite assessment over 3 consecutive days at the end of the ML and EL diets.

(B) Temporal appetite scores based on hourly VAS (0–100 mm) assessments across 3 consecutive days at the end of the free-living ML and EL diets. First VAS completed upon waking and then hourly until bedtime.

(C) Temporal appetite scores during the in-lab test day. VAS completed immediately prior to breakfast and then every 30 min for the remainder of the day. First 6 h post-breakfast was spent in the human nutrition unit facility; thereafter, participants returned to their own free-living context and resumed meals at their own predetermined times.

Appetite score calculated as (hunger + [100 – fullness] + prospective consumption + desire to eat)/4. Values displayed as mean ± SEM, *$p < 0.05$ between ML and EL diets. EL, evening loaded; ML, morning loaded; VAS, visual analog scale.

emptying can influence feelings of fullness when early feeding is applied. This is a useful feature of morning eating, particularly when participants are in a negative energy balance, in order to promote adherence to a calorie-controlled diet. Diets that promote greater calorie intake in the morning and lower calorie intake (or longer fasting) in the evening are likely to produce sustained weight loss (Parr et al., 2020).

Distribution of calorie intake does not affect EE

A key finding of this intervention was that the ML diet did not alter EE in comparison to the isocaloric EL diet. We controlled calorie intake to explicitly measure the effects on all components of total daily EE (TDEE) in a free-living lifestyle setting. Figure 2B demonstrates no difference in TDEE between the ML and EL feeding regimes (2,871 versus 2,846 kcal/day, respectively; SED 100.7 kcal, $p = 0.184$), which represents 1.7 and 1.68 physical activity level (TDEE/RMR) for the ML and EL diets, respectively. This is measured objectively with DLW during the 4-week weight-loss diets. DLW is the gold standard approach to measuring TDEE outside the lab (Speakman et al., 2021). This enabled the most accurate assessment of any differences arising in TDEE as a function of daily energy distribution. While some previous studies have reported greater weight loss with morning or "early" calorie intake in contrast to evening or "later" calorie intake, these studies did not include accurate assessment of EE (Garaulet et al., 2013; Jakubowicz et al., 2013). Here we clearly demonstrate that calorie utilization does not vary with time of day, suggesting that metabolic adaptation does not provide the basis for the enhanced weight loss associated with morning calorie loading seen in other studies. Instead, we suggest that the changes in weight loss seen in prior work are likely to be behavioral (e.g., changes in appetite) with the results reported being due to variation and misreporting of EI. This is in agreement with acute/short-term studies (24 h to 6 days) involving breakfast skipping, whereby skipping breakfast (and thereby pushing calorie intake later into the day) had no effect on TDEE measured in a respiratory chamber (Kobayashi et al., 2014; Nas et al., 2017; Ogata et al., 2019). Similarly, Ravussin et al. (2019) found no differences in TDEE in a respiratory chamber when participants undertook either early TRF (eating from 8 a.m. to 2 p.m.) or control schedule (eating from 8 a.m. to 8 p.m.) for 4 days each. However, although respiratory chamber studies allow for extremely rigorous control over calorie intake and assessment of EE, they are restricted to very short durations of measurement and largely impede on an individual's normal physical activity. In contrast, the DLW method we applied allowed for assessment of TDEE over the entire ML and EL interventions in a real-life setting.

In this study, we measured and report all components of TDEE (Figure 2; Table 2), including measures of RMR, TEF, and physical activity with actigraphy, to isolate the distinct area of metabolic function that may have contributed to differences in energy balance reported in prior studies. As anticipated, RMR was significantly lower after both the ML and EL weight-loss diets compared to baseline, corresponding with a lower body weight (Figure 2C). This was expected as many studies have reported

CellPress
OPEN ACCESS

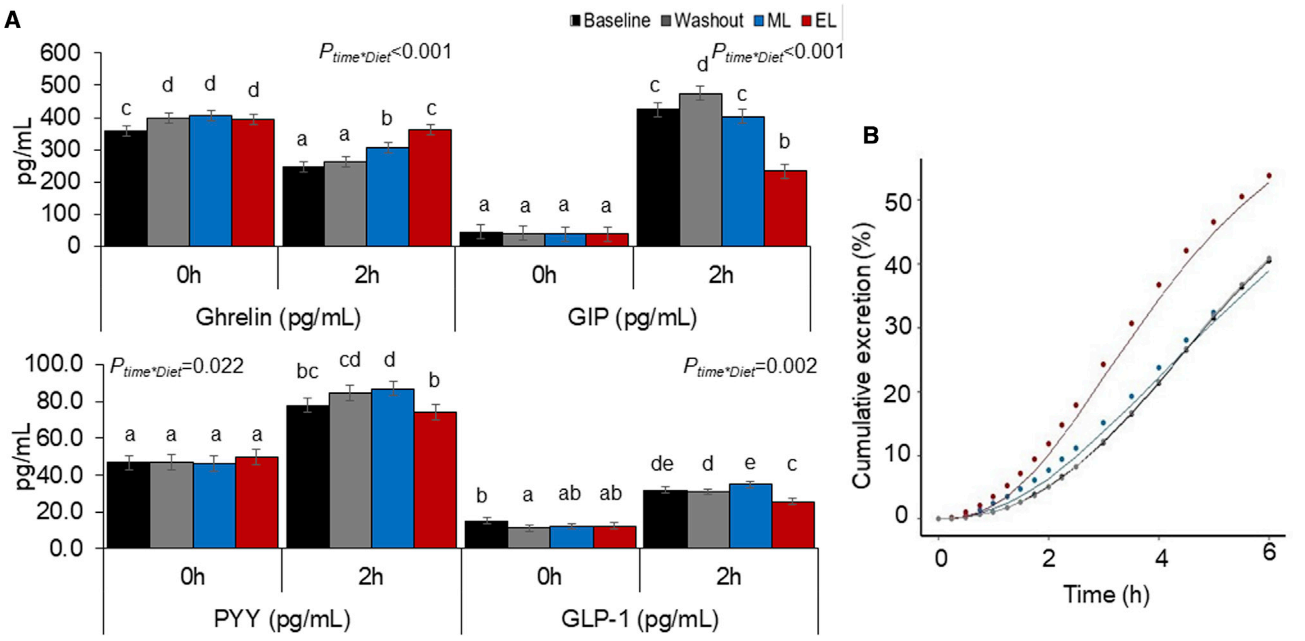

Figure 4. The large morning meal on the ML diet resulted in greater changes in appetite hormones (suppression of hunger hormone ghrelin and increase in satiety hormones) and slower gastric emptying compared to the smaller morning meal on the EL diet

(A) Appetite hormones measured at the end of each diet phase. Samples collected before, 0 h, and 2 h after the breakfast meal. Values expressed as mean ± SEM. Bars with different letters are significantly different ($p < 0.05$).

(B) Gastric emptying measured using ^{13}C octanoic acid breath test shown as cumulative excretion of contents from the stomach over time.

EL, evening loaded; ML, morning loaded.

a reduction in RMR linked to reduced body mass and changes in body composition (Coutinho et al., 2018; Martins et al., 2020). However, most importantly, there were no differences in RMR between the ML or EL diets. This is consistent with the results of Versteeg et al. (2018), who found no differences in RMR in obese men after 4 weeks of following ML versus EL hypocaloric diets. Similarly, other RCTs demonstrate no metabolic adaption (change in RMR) to breakfast feeding in comparison to breakfast skipping (Betts et al., 2014) in lean adults. Interestingly, this study by Betts et al. (2014) found that although there were no changes in RMR, physical activity was significantly higher with breakfast consumption, albeit no differences in weight change occurred between groups, likely due to higher calorie intake in the breakfast group. Other groups have reported similar findings with breakfast consumption linked to higher levels of physical activity and lower sedentary time (Corder et al., 2014; Zakrzewski-Fruer et al., 2019), possibly acting as a contributing factor to differential energy balance with ML or EL calorie intake. It has previously been summarized that while breakfast inclusion does not seem to affect basal metabolism, breakfast omission may influence free-living physical activity and endurance exercise performance throughout the day (Clayton and James, 2016). We report no differences in physical activity, measured across the 3 days at the end of each diet. It may be that simply wearing the devices encouraged participants to be more active and obscured any true differences. However, given the lack of differences in TDEE measured over the entire 4 weeks, this is unlikely to be the case. Longer-term studies are required to understand the impact of meal timing and daily calorie distribution on longer-term exercise training and incidental activity in both lean and overweight subjects.

Other studies have suggested that diurnal variation in TEF may also contribute to energy imbalance, with proposed lower evening TEF contributing to lower weight loss with EL calorie intake (Bo et al., 2015; Morris et al., 2015; Richter et al., 2020; Romon et al., 1993). Due to feasibility, we were only able to measure TEF following the morning meal, and hence meal size largely dictated the response, with TEF being significantly larger after the larger ML breakfast (147 kcal) in contrast to the smaller EL breakfast (98 kcal). We recently reported (Ruddick-Collins et al., 2022) that differences in TEF between morning and evening feeding can be explained by underlying circadian RMR, which is independent of an acute effect of eating. As a consequence, lower evening TEF may be more apparent than real given that evening TEF is suppressed but extended, thereby continuing into the night and causing an apparently higher sleeping metabolic rate. Given we were unable to detect any differences in TDEE or weight change between the ML and EL diet regimes, we believe that any variation in TEF from ML and EL dietary regimes has a negligible impact on energy balance. In summary, we were not able to detect any differences in TDEE or any of the components of TDEE linked to differential calorie loading. Our study is the first controlled diet study to precisely measure all components of TDEE in order to directly assess the effects of morning and evening calorie loading. Our data show that differences in EE do not contribute to differences in energy balance and weight loss with ML calorie intake over a 4-week period in healthy subjects with obesity. Our study supports and expands

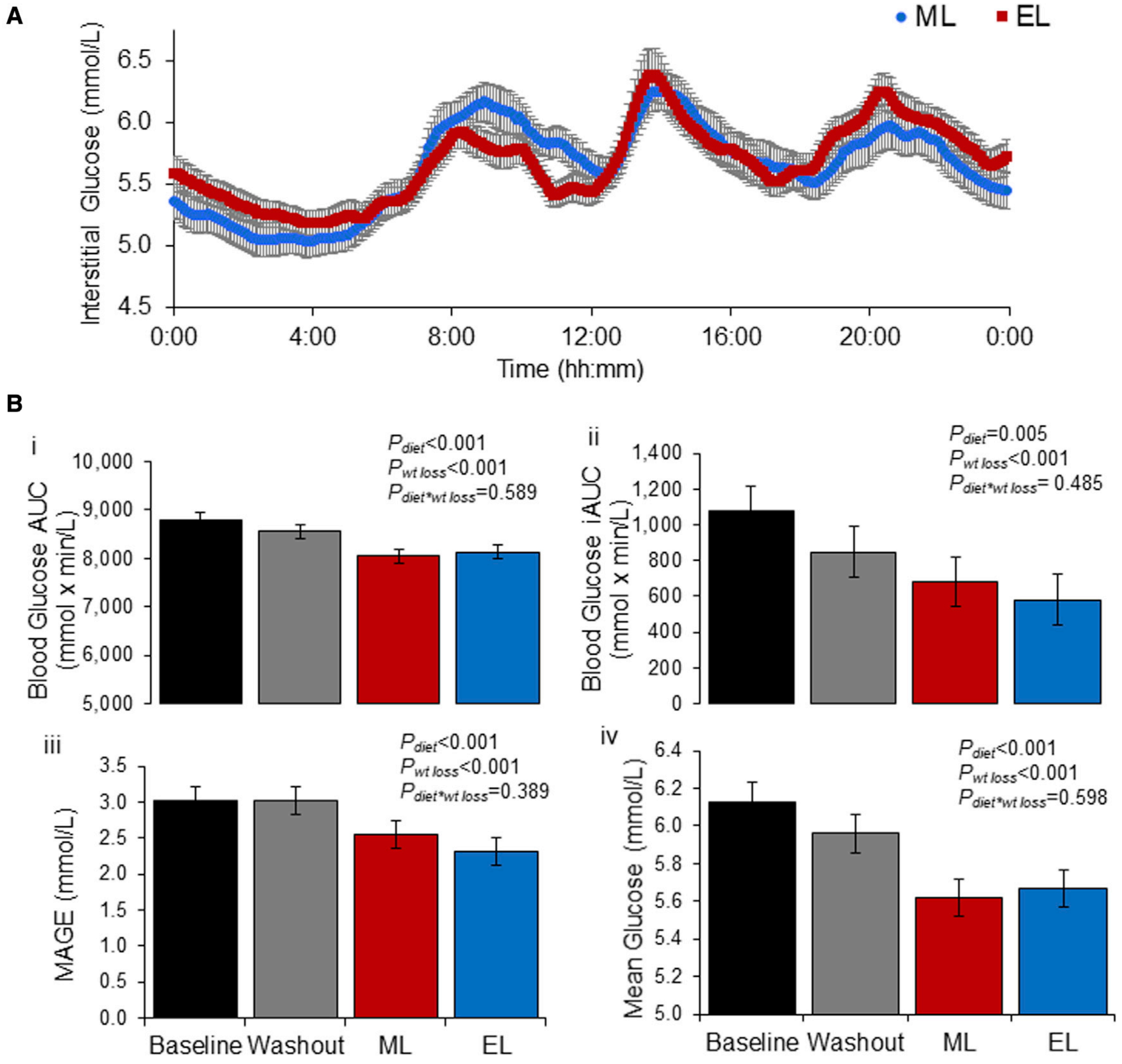

Figure 5. Interstitial glucose measured using CGMs for 3 days at the end of each diet phase

(A) 24 h temporal profile of interstitial glucose during the ML and EL diets. Interstitial glucose was measured every 10 s and average glucose values stored for each 5 min period after midnight. Data shown as the average for each 5 min period over 3 consecutive days of measurements.

(B) The data from the 3 consecutive days was used to calculate 24 h glucose metrics: (1) AUC, (2) iAUC, (3) MAGE, and (4) mean.

Values displayed as mean ± SEM. AUC, area under the curve; EL, evening loaded; iAUC, incremental area under the curve; MAGE, mean amplitude of glycemic excursions; ML, morning loaded.

on findings by Ravussin et al. (2019), who reported no effect of early TRF on TDEE, measured for 24 h in a respiratory chamber. Interestingly, these authors suggest that meal-timing interventions likely facilitate weight loss through impact on appetite rather than EE. While our data accord with this conclusion, a recent systematic review has highlighted the potential unreliability of subjective appetite ratings to predict measurable changes in EI (Holt et al., 2017). Thus, the physiological relevance on overall energy balance of the differences in subjective appetite seen in this study remain to be rigorously assessed.

ML and EL weight-loss diets result in similar changes in glucose, insulin, and blood lipid profiles

We applied continuous glucose monitoring (CGM) for 3 consecutive days at the end of each dietary phase to assess glycemic response. There was a significant effect of weight loss on all metrics calculated from CGM (Figure 5; Table S5). Weight loss resulted in significantly lower mean daily glucose, incremental and absolute area under the curve, maximum glucose levels, and daily glucose variability (variability assessed with MAGE [mean amplitude of glycemic excursions] (Monnier et al., 2008; Service et al., 1970) and SD). Interestingly, when the CGM data were analyzed and defined by diet and clock time as distinct 4 h time blocks (12 midnight–4 a.m., 4–8 a.m., 8–12 noon, 12 noon–4 p.m., 4–8 p.m., and 8–12 midnight), there is only a significantly higher postprandial rise in interstitial glucose for the EL diet, between 8 and 12 p.m. (p = 0.012), when compared to the ML regime. This reflects the greater meal size (and glycemic load) with the larger dinner meal, but interestingly, there is no reciprocal statistical difference in the morning period when a large breakfast is consumed. All CGM data are

presented in Figure S2, showing a decline in interstitial glucose in both weight-loss diets, linked to reduced body mass and calorie intake, relative to baseline. There is a known circadian impact on glucose regulation, with poorer glucose control in the evening in comparison to the morning, despite identical meals and comparable fasting times (Grant et al., 2017; Morgan et al., 1999). Weight loss is well known to improve metabolic control (Wilding, 2014), even in healthy subjects with obesity (Anderson et al., 2003). The effect of time of eating and calorie loading is less well documented. Similar to our study, Hutchison et al. (2019) report no mealtime interaction on CGM glucose parameters in healthy subjects with obesity, when randomized to early or late feeding (eTRF or dTRF). Both TRF regimes improved glucose tolerance, but there was no mealtime interaction. It is important to note that participants in our study were required to have normal glycemic control prior to commencing the study, and results may differ in subjects with type 2 diabetes. Plasma measures of glucose and insulin parameters further supported these results, with reduced glucose and significantly lower fasting insulin and HOMA following both calorie restriction diets, irrespective of ML or EL calorie loading (Table 2). Similarly, compared to baseline both the ML and EL diets resulted in significant yet comparable reductions in total cholesterol, LDLs, and triglycerides (Tables 2 and S6). There were no mealtime effects on metabolic parameters, and all significant effects relate to weight loss. It is possible that the benefit of weight loss outweighed any differences arising from calorie distribution or that evening meals were not large or late enough to cause the negative impacts seen in other studies.

Conclusion

To our knowledge, this is the first randomized cross-over trial comparing ML and EL calorie intake, which includes such comprehensive assessment of both EI and EE. Our study sought to determine the impact of daily calorie distribution on all components of EE as well as factors influencing calorie intake and metabolic health. Our study disproves earlier studies that infer time-of-day calorie intake may influence energy balance through metabolic adaptation and instead implies that changes in appetite may be involved in improving weight loss with ML EI. Although our study controlled calorie intake, the greater satiety and lower hunger on the ML diet may contribute to behavioral changes (reduced calorie intake later in the day), which would support greater weight loss. While we did not find significant effects of calorie distribution on metabolic health parameters, other factors may have influenced these results: for example, the inclusion of "healthy" overweight subjects who were otherwise metabolically healthy individuals. Thus, further studies are needed to explore mealtime effects on various population groups and with greater manipulation of meal timing. In this population, ML feeding is likely to improve weight-loss success through behavioral adaptions (reduced calorie intake and dietary compliance) as a result of greater suppression of appetite across the day. Nonetheless, whether reduced appetite translates into reduced EI beyond the conditions tested in this study remains to be demonstrated.

Limitations of study

We acknowledge a number of limitations in this study. The trial was conducted under free-living conditions. While the benefit of this is the replication of real-living context (i.e., living in a lab would largely impact participants' normal physical activity), this increases the chances of participant noncompliance. However, given the regularity of attendance at the nutrition unit, provision of all meals, and regular body weight checks, we believe any noncompliance not reported in food records was minimal and did not largely influence the study outcomes. Although participants were shown meal items during their screening to ensure they could eat required items and were encouraged to consume their entire meals, many were unable to eat the full quantity provided and/or refused to consume certain items they did not enjoy. Feeling fuller after switching to different meal plans can often occur despite calorie restriction due to more satiating foods (e.g., high protein, high volume, high fiber). Also, while the study aimed for 45% of calories to be consumed at breakfast (ML) or dinner (EL), the sheer size of the meals resulted in some participants being physically unable to consume the entire meals. Although other studies report 50% of calories being consumed within a single meal, they do not specifically report actual calorie intake (only the prescribed intake) (Jakubowicz et al., 2013), or intake was provided as liquid meal (Versteeg et al., 2018), which may have been easier for participants to consume but does not represent most real-world diets. Due to feasibility, we were also only able to measure certain metabolic responses following the breakfast meal. There is the possibility that differences in metabolic health parameters such as plasma glucose, insulin, and fatty acids, as well as TEF, may have been evident following the evening meal. The inclusion of a glucose or insulin clamp technique would have been a more robust measure of glucose homeostasis, rather than the application of CGM in the current study, which only measures interstitial glucose. It would be interesting to see these advanced techniques applied during different times of the day to assess chrono-nutrition influence on metabolic health. This is supported by the current CGM data, which suggest that the evening period before sleep (8 p.m.–12 midnight) is an interesting time period to further assess the impact of timing of eating and fasting within the context of TRE to improve glucose homeostasis in the absence of weight loss.

The W period in the current study was only a week, and this may not have been long enough to reduce the influence of the previous diet period on the next assessment period. A within-subject cross-over design was necessary to assess subjective changes in appetite but has a higher participant burden due to the length of time on a controlled diet (Lichtenstein et al., 2021). Furthermore, if we consider the measured TDEE/RMR during the weight loss phases, this indicated participants likely had a physical activity level value greater than 1.5. However, the measured body weight change during the W period was +0.29 kg, which was within normal daily variation and suggests that the group was held near energy balance. It has been suggested that the energy content of weight loss is approximately 3,500 kcal per pound of body weight, and the work of Hall (2008) and Hall et al. (2011) has challenged this, highlighted modeling of tissue change during weight loss. In our study, the subjects lost between 3% and 4% body weight during each 4-week diet period, and the application of gold standard techniques allowed some comparative mathematical modeling and commentary on the group-level data to assess the energy cost of energy balance. In summary, all the data from body mass (kg tissue) change, energy balance (EE by DLW and EI by food

intake), and body composition (4-compartment model) are in agreement that the weight loss was similar between the ML and EL diet phases. This can be considered in different ways:

- Cumulative energy imbalance (calculated from loss of fat and protein mass body composition) over 28 days is −28,335 kcal (ML), which is an average negative energy balance of 1,012 kcal per day. For the EL diet, the cumulative imbalance for 28 days was 28,212 kcal (from body composition; data shown in Figure S2), which is 1,008 kcal per day.
- Calculated negative energy deficit from DLW-derived EE averaged over the 28 days (with the assumption it was constant) and EI from food records for the ML diet period is 1,736 EI and 2,871 EE, leaving a daily energy deficit of 1,135 kcal per day. Similarly, it was 1,749 EI and 2,846 EE for the EL diet period, leaving a daily energy deficit of 1,097 kcal per day.

These calculations suggest there is a discrepancy of 90–100 kcal per day, similar for both diet treatments, which could mean the subjects consumed more energy than we thought (and did not report this) or represents the error of modeling daily small changes in energy balance with these techniques. A longer study to achieve at least 5% (ideally 10%) weight loss and a larger (n) study would be able to achieve this degree of energy cost of weight-loss modeling with a similar approach.

Participants within our study were also able to self-select their meals times. Although participants were asked to maintain these across the study and there were only small differences between the ML and EL diets, on average participants consumed their meals within a <12 h window. The modest (<30 min) increase in average daily dinner-to-breakfast fast in the EL condition could potentially have influenced our measures before and after breakfast. Any such influence would be negligible, though, given (1) the small difference within the overall (ca. 13 h) average dinner-to-breakfast fasting window and (2) the prolonged post-prandial response expected after dinner in the EL condition. Despite this, we may have seen more pronounced effects if participants had a longer feeding window across the day (e.g., it has been shown that many people eat over as much as 14 h per day; Gill and Panda, 2015; Jefcoate et al., 2021), or if the large dinner meal was consumed later in the evening closer to bed time. It has been reported that eating closer to dim-light melatonin onset is in itself a predictor of higher BMI (McHill et al., 2017). Overall, our trial used many gold standard techniques to effectively measure EI and all areas of EE, and therefore the outcomes from our study are highly thorough. However, further research is needed to understand if ML feeding produces superior metabolic outcomes depending on the eating window and timing of the evening meal, and to assess all aspects of energy metabolism after both breakfast and dinner meals.

STAR★METHODS

Detailed methods are provided in the online version of this paper and include the following:

- KEY RESOURCES TABLE
- RESOURCE AVAILABILITY
  - Lead contact
  - Materials availability
  - Data and code availability
- EXPERIMENTAL MODEL AND SUBJECT DETAILS
- METHOD DETAILS
  - Study design
  - Formulation and preparation of the diets
  - Measurement of anthropometric variables
  - Total daily energy expenditure by doubly labelled water ($^2H_2$ ^{18}O stable isotopes)
  - Resting metabolic rate (RMR) and thermic effect of food (TEF)
  - Assessment of gastric emptying
  - Assessment of subjective appetite
  - Metabolic profile and glucose homeostasis
  - Continuous glucose monitoring
  - Sleep record
  - Physical activity
- QUANTIFICATION AND STATISTICAL ANALYSIS
  - Power analysis
  - Statistical analysis

SUPPLEMENTAL INFORMATION

Supplemental information can be found online at https://doi.org/10.1016/j.cmet.2022.08.001.

ACKNOWLEDGMENTS

The authors gratefully acknowledge Sylvia Stephen, Jean Bryce, Nina Lamza, Karen Taylor, Melanie Hudson, Kat Niblock, Ewa Wojtaczka, Aimee Sutherland, David Bremner, Claire Kidd, and Alicia Bryce at the Human Nutrition Unit of the Rowett Institute for their support in meal preparation and participant assessment. The authors acknowledge the contribution of NIHR Core Biochemistry Assay Laboratory, Cambridge Biomedical Research Centre (gut hormone analysis), and Loek Wouters at Maastricht University, Netherlands (DLW analysis). The authors also gratefully acknowledge Claus-Dieter Mayer for statistical analysis and modeling of the gastric emptying data. The authors gratefully acknowledge funding from the Medical Research Council (grant MR/P012205/1, The Big Breakfast study). A.M.J., P.J.M., G.W.H., and J.A.N.F. also acknowledge funding support from the Scottish Government, Rural and Environment Science and Analytical Services Division.

AUTHOR CONTRIBUTIONS

Conceptualization, design, and funding acquisition, A.M.J., P.J.M., and J.D.J.; investigation, L.C.R.-C. and C.L.F.; DLW analysis and modeling, K.R.W.; statistical analysis, G.W.H. and J.A.N.F.; writing – original draft, L.C.R.-C. and A.M.J.; writing – review & editing, all authors.

DECLARATION OF INTERESTS

J.D.J. has collaborated with Nestlé and has previously undertaken consultancy work for Kellogg's.

Received: December 21, 2021
Revised: May 23, 2022
Accepted: August 2, 2022
Published: September 9, 2022

REFERENCES

Almoosawi, S., Winter, J., Prynne, C.J., Hardy, R., and Stephen, A.M. (2012). Daily profiles of energy and nutrient intakes: are eating profiles changing over time? Eur. J. Clin. Nutr. *66*, 678–686.

Anderson, G.H., Catherine, N.L.A., Woodend, D.M., and Wolever, T.M.S. (2002). Inverse association between the effect of carbohydrates on blood glucose and subsequent short-term food intake in young men. Am. J. Clin. Nutr. *76*, 1023–1030.

Anderson, J.W., Kendall, C.W.C., and Jenkins, D.J.A. (2003). Importance of weight management in type 2 diabetes: review with meta-analysis of clinical studies. J. Am. Coll. Nutr. *22*, 331–339.

Arble, D.M., Bass, J., Laposky, A.D., Vitaterna, M.H., and Turek, F.W. (2009). Circadian timing of food intake contributes to weight gain. Obesity *17*, 2100–2102.

Baecke, J.A., Burema, J., and Frijters, J.E. (1982). A short questionnaire for the measurement of habitual physical activity in epidemiological studies. Am. J. Clin. Nutr. *36*, 936–942.

Beaulieu, K., Oustric, P., Alkahtani, S., Alhussain, M., Pedersen, H., Quist, J.S., Færch, K., and Finlayson, G. (2020). Impact of meal timing and chronotype on food reward and appetite control in young adults. Nutrients *12*, 1506.

Belkacemi, L., Selselet-Attou, G., Hupkens, E., Nguidjoe, E., Louchami, K., Sener, A., and Malaisse, W.J. (2012). Intermittent fasting modulation of the diabetic syndrome in streptozotocin-injected rats. Int. J. Endocrinol. *2012*, 962012.

Betts, J.A., Richardson, J.D., Chowdhury, E.A., Holman, G.D., Tsintzas, K., and Thompson, D. (2014). The causal role of breakfast in energy balance and health: a randomized controlled trial in lean adults. Am. J. Clin. Nutr. *100*, 539–547.

Black, A.E., Prentice, A.M., and Coward, W.A. (1986). Use of food quotients to predict respiratory quotients for the doubly-labelled water method of measuring energy expenditure. Hum. Nutr. Clin. Nutr. *40*, 381–391.

Bo, S., Fadda, M., Castiglione, A., Ciccone, G., De Francesco, A., Fedele, D., Guggino, A., Parasiliti Caprino, M., Ferrara, S., Vezio Boggio, M., et al. (2015). Is the timing of caloric intake associated with variation in diet-induced thermogenesis and in the metabolic pattern? A randomized cross-over study. Int. J. Obes. *39*, 1689–1695.

Brouwer, E. (1957). On simple formulae for calculating the heat expenditure and the quantities of carbohydrate and fat oxidized in metabolism of men and animals, from gaseous exchange (oxygen intake and carbonic acid output) and urine-N. Acta Physiol. Pharmacol. Neerl. *6*, 795–802.

Buysse, D.J., Reynolds, C.F.,, 3rd, Kupfer, D.J., Monk, T.H., Berman, S.R., and Kupfer, D.J. (1989). The Pittsburgh Sleep Quality Index: a new instrument for psychiatric practice and research. Psychiatry Res. *28*, 193–213.

Carlson, O., Martin, B., Stote, K.S., Golden, E., Maudsley, S., Najjar, S.S., Ferrucci, L., Ingram, D.K., Longo, D.L., Rumpler, W.V., et al. (2007). Impact of reduced meal frequency without caloric restriction on glucose regulation in healthy, normal-weight middle-aged men and women. Metabolism *56*, 1729–1734.

Cienfuegos, S., Gabel, K., Kalam, F., Ezpeleta, M., Wiseman, E., Pavlou, V., Lin, S., Oliveira, M.L., and Varady, K.A. (2020). Effects of 4-and 6-h time-restricted feeding on weight and cardiometabolic health: a randomized controlled trial in adults with obesity. Cell Metab. *32*, 366–378.e3.

Clayton, D.J., and James, L.J. (2016). The effect of breakfast on appetite regulation, energy balance and exercise performance. Proc. Nutr. Soc. *75*, 319–327.

Corder, K., van Sluijs, E.M.F., Ridgway, C.L., Steele, R.M., Prynne, C.J., Stephen, A.M., Bamber, D.J., Dunn, V.J., Goodyer, I.M., and Ekelund, U. (2014). Breakfast consumption and physical activity in adolescents: daily associations and hourly patterns. Am. J. Clin. Nutr. *99*, 361–368.

Coutinho, S.R., With, E., Rehfeld, J.F., Kulseng, B., Truby, H., and Martins, C. (2018). The impact of rate of weight loss on body composition and compensatory mechanisms during weight reduction: a randomized control trial. Clin. Nutr. *37*, 1154–1162.

de Castro, J.M. (1987). Circadian rhythms of the spontaneous meal pattern, macronutrient intake, and mood of humans. Physiol. Behav. *40*, 437–446.

de Castro, J.M. (2004). The time of day of food intake influences overall intake in humans. J. Nutr. *134*, 104–111.

Elia, M., and Livesey, G. (1992). Energy expenditure and fuel selection in biological systems: the theory and practice of calculations based on indirect calorimetry and tracer methods. World Rev. Nutr. Diet. *70*, 68–131.

Finglas, P., Roe, M., Pinchen, H., Berr, R., Church, S., Dodhia, S., Farron-Wilson, M., and Swan, G. (2014). McCance and Widdowson's the Composition of Foods (Royal Society of Chemistry).

Flanagan, A., Bechtold, D.A., Pot, G.K., and Johnston, J.D. (2021). Chrono-nutrition: from molecular and neuronal mechanisms to human epidemiology and timed feeding patterns. J. Neurochem. *157*, 53–72.

Flint, A., Raben, A., Blundell, J.E., and Astrup, A. (2000). Reproducibility, power and validity of visual analogue scales in assessment of appetite sensations in single test meal studies. Int. J. Obes. Relat. Metab. Disord. *24*, 38–48.

Fonken, L.K., Workman, J.L., Walton, J.C., Weil, Z.M., Morris, J.S., Haim, A., and Nelson, R.J. (2010). Light at night increases body mass by shifting the time of food intake. Proc. Natl. Acad. Sci. USA *107*, 18664–18669.

Fuller, N.J., Jebb, S.A., Laskey, M.A., Coward, W.A., and Elia, M. (1992). Four-component model for the assessment of body composition in humans: comparison with alternative methods, and evaluation of the density and hydration of fat-free mass. Clin. Sci. *82*, 687–693.

Garaulet, M., and Gómez-Abellán, P. (2014). Timing of food intake and obesity: a novel association. Physiol. Behav. *134*, 44–50.

Garaulet, M., Gómez-Abellán, P., Alburquerque-Béjar, J.J., Lee, Y.C., Ordovás, J.M., and Scheer, F.A.J.L. (2013). Timing of food intake predicts weight loss effectiveness. Int. J. Obes. *37*, 604–611.

Ghoos, Y.F., Maes, B.D., Geypens, B.J., Mys, G., Hiele, M.I., Rutgeerts, P.J., and Vantrappen, G. (1993). Measurement of gastric emptying rate of solids by means of a carbon-labeled octanoic acid breath test. Gastroenterology *104*, 1640–1647.

Gill, S., and Panda, S. (2015). A smartphone app reveals erratic diurnal eating patterns in humans that can be modulated for health benefits. Cell Metab. *22*, 789–798.

Goo, R.H., Moore, J.G., Greenberg, E., and Alazraki, N.P. (1987). Circadian variation in gastric emptying of meals in humans. Gastroenterology *93*, 515–518.

Grant, C.L., Coates, A.M., Dorrian, J., Kennaway, D.J., Wittert, G.A., Heilbronn, L.K., Pajcin, M., Della Vedova, C., Gupta, C.C., and Banks, S. (2017). Timing of food intake during simulated night shift impacts glucose metabolism: A controlled study. Chronobiol. Int. *34*, 1003–1013.

Hall, K.D. (2008). What is the required energy deficit per unit weight loss? Int. J. Obes. *32*, 573–576.

Hall, K.D., Sacks, G., Chandramohan, D., Chow, C.C., Wang, Y.C., Gortmaker, S.L., and Swinburn, B.A. (2011). Quantification of the effect of energy imbalance on bodyweight. Lancet *378*, 826–837.

Hatori, M., Vollmers, C., Zarrinpar, A., DiTacchio, L., Bushong, E.A., Gill, S., Leblanc, M., Chaix, A., Joens, M., Fitzpatrick, J.A.J., et al. (2012). Time-restricted feeding without reducing caloric intake prevents metabolic diseases in mice fed a high-fat diet. Cell Metab. *15*, 848–860.

Haycock, G.B., Schwartz, G.J., and Wisotsky, D.H. (1978). Geometric method for measuring body surface area: a height-weight formula validated in infants, children, and adults. J. Pediatr. *93*, 62–66.

Holt, G.M., Owen, L.J., Till, S., Cheng, Y., Grant, V.A., Harden, C.J., and Corfe, B.M. (2017). Systematic literature review shows that appetite rating does not predict energy intake. Crit. Rev. Food Sci. Nutr. *57*, 3577–3582.

Horne, J.A., and Östberg, O. (1976). A self-assessment questionnaire to determine morningness-eveningness in human circadian rhythms. Int. J. Chronobiol. *4*, 97–110.

Hutchison, A.T., Regmi, P., Manoogian, E.N.C., Fleischer, J.G., Wittert, G.A., Panda, S., and Heilbronn, L.K. (2019). Time-restricted feeding improves glucose tolerance in men at risk for type 2 diabetes: a randomized crossover trial. Obesity *27*, 724–732.

Jakubowicz, D., Barnea, M., Wainstein, J., and Froy, O. (2013). High caloric intake at breakfast vs. dinner differentially influences weight loss of overweight and obese women. Obesity *21*, 2504–2512.

Jefcoate, P.W., Robertson, M.D., Ogden, J., and Johnston, J.D. (2021). Identification of factors influencing motivation to undertake time-restricted feeding in humans. Appetite *164*, 105240.

Johns, M.W. (1991). A new method for measuring daytime sleepiness: the Epworth sleepiness scale. Sleep *14*, 540–545.

Johnstone, A.M., Murison, S.D., Duncan, J.S., Rance, K.A., and Speakman, J.R. (2005). Factors influencing variation in basal metabolic rate include fat-free mass, fat mass, age, and circulating thyroxine but not sex, circulating leptin, or triiodothyronine. Am. J. Clin. Nutr. *82*, 941–948.

Johnstone, A.M., Rance, K.A., Murison, S.D., Duncan, J.S., and Speakman, J.R. (2006). Additional anthropometric measures may improve the predictability of basal metabolic rate in adult subjects. Eur. J. Clin. Nutr. *60*, 1437–1444.

Johnstone, A.M., Kelly, J., Ryan, S., Romero-Gonzalez, R., McKinnon, H., Fyfe, C., Naslund, E., Lopez-Nicolas, R., Bosscher, D., et al. (2020). Nondigestible carbohydrates affect metabolic health and gut microbiota in overweight adults after weight loss. J. Nutr. *150*, 1859–1870.

Kobayashi, F., Ogata, H., Omi, N., Nagasaka, S., Yamaguchi, S., Hibi, M., and Tokuyama, K. (2014). Effect of breakfast skipping on diurnal variation of energy metabolism and blood glucose. Obes. Res. Clin. Pract. *8*, e201–e298.

Lichtenstein, A.H., Petersen, K., Barger, K., Hansen, K.E., Anderson, C.A.M., Baer, D.J., Lampe, J.W., Rasmussen, H., and Matthan, N.R. (2021). Perspective: design and conduct of human nutrition randomized controlled trials. Adv. Nutr. *12*, 4–20.

Marfell-Jones, M.J., Stewart, A., and De Ridder, J. (2012). International Standards for Anthropometric Assessment (International Society for the Advancement of Kinanthropometry).

Martins, C., Roekenes, J., Salamati, S., Gower, B.A., and Hunter, G.R. (2020). Metabolic adaptation is an illusion, only present when participants are in negative energy balance. Am. J. Clin. Nutr. *112*, 1212–1218.

Matthews, D.R., Hosker, J.P., Rudenski, A.S., Naylor, B.A., Treacher, D.F., and Turner, R.C. (1985). Homeostasis model assessment: insulin resistance and β-cell function from fasting plasma glucose and insulin concentrations in man. Diabetologia *28*, 412–419.

McHill, A.W., Phillips, A.J., Czeisler, C.A., Keating, L., Yee, K., Barger, L.K., Garaulet, M., Scheer, F.A., and Klerman, E.B. (2017). Later circadian timing of food intake is associated with increased body fat. Am. J. Clin. Nutr. *106*, 1213–1219.

Monnier, L., Colette, C., and Owens, D.R. (2008). Glycemic variability: the third component of the dysglycemia in diabetes. Is it important? How to measure it? J. Diabetes Sci. Technol. *2*, 1094–1100.

Morgan, L.M., Aspostolakou, F., Wright, J., and Gama, R. (1999). Diurnal variations in peripheral insulin resistance and plasma non-esterified fatty acid concentrations: a possible link? Ann. Clin. Biochem. *36*, 447–450.

Moro, T., Tinsley, G., Bianco, A., Marcolin, G., Pacelli, Q.F., Battaglia, G., Palma, A., Gentil, P., Neri, M., and Paoli, A. (2016). Effects of eight weeks of time-restricted feeding (16/8) on basal metabolism, maximal strength, body composition, inflammation, and cardiovascular risk factors in resistance-trained males. J. Transl. Med. *14*, 290.

Morris, C.J., Garcia, J.I., Myers, S., Yang, J.N., Trienekens, N., and Scheer, F.A.J.L. (2015). The human circadian system has a dominating role in causing the morning/evening difference in diet-induced thermogenesis. Obesity *23*, 2053–2058.

Nas, A., Mirza, N., Hägele, F., Kahlhöfer, J., Keller, J., Rising, R., Kufer, T.A., and Bosy-Westphal, A. (2017). Impact of breakfast skipping compared with dinner skipping on regulation of energy balance and metabolic risk. Am. J. Clin. Nutr. *105*, 1351–1361.

Ogata, H., Kayaba, M., Tanaka, Y., Yajima, K., Iwayama, K., Ando, A., Park, I., Kiyono, K., Omi, N., Satoh, M., and Tokuyama, K. (2019). Effect of skipping breakfast for 6 days on energy metabolism and diurnal rhythm of blood glucose in young healthy Japanese males. Am. J. Clin. Nutr. *110*, 41–52.

Olsen, M.K., Choi, M.H., Kulseng, B., Zhao, C.M., and Chen, D. (2017). Time-restricted feeding on weekdays restricts weight gain: a study using rat models of high-fat diet-induced obesity. Physiol. Behav. *173*, 298–304.

Parr, E.B., Heilbronn, L.K., and Hawley, J.A. (2020). A time to eat and a time to exercise. Exerc. Sport Sci. Rev. *48*, 4–10.

Qian, J., Morris, C.J., Caputo, R., Garaulet, M., and Scheer, F.A.J.L. (2019). Ghrelin is impacted by the endogenous circadian system and by circadian misalignment in humans. Int. J. Obes. *43*, 1644–1649.

Ravussin, E., Beyl, R.A., Poggiogalle, E., Hsia, D.S., and Peterson, C.M. (2019). Early time-restricted feeding reduces appetite and increases fat oxidation but does not affect energy expenditure in humans. Obesity *27*, 1244–1254.

Reed, G.W., and Hill, J.O. (1996). Measuring the thermic effect of food. Am. J. Clin. Nutr. *63*, 164–169.

Richter, J., Herzog, N., Janka, S., Baumann, T., Kistenmacher, A., and Oltmanns, K.M. (2020). Twice as high diet-induced thermogenesis after breakfast vs dinner on high-calorie as well as low-calorie meals. J. Clin. Endocrinol. Metab. *105*, e211–e221.

Roenneberg, T., Wirz-Justice, A., and Merrow, M. (2003). Life between clocks: daily temporal patterns of human chronotypes. J. Biol. Rhythms *18*, 80–90.

Roenneberg, T., Kuehnle, T., Juda, M., Kantermann, T., Allebrandt, K., Gordijn, M., and Merrow, M. (2007). Epidemiology of the human circadian clock. Sleep Med. Rev. *11*, 429–438.

Romon, M., Edme, J.L., Boulenguez, C., Lescroart, J.L., and Frimat, P. (1993). Circadian variation of diet-induced thermogenesis. Am. J. Clin. Nutr. *57*, 476–480.

Ruddick-Collins, L.C., Morgan, P.J., and Johnstone, A.M. (2020). Mealtime: a circadian disruptor and determinant of energy balance? J. Neuroendocrinol. *32*, e12886.

Ruddick-Collins, L.C., Flanagan, A., Johnston, J.D., Morgan, P.J., and Johnstone, A.M. (2022). Circadian rhythms in resting metabolic rate account for apparent daily rhythms in the thermic effect of food. J. Clin. Endocrinol. Metab. *107*, e708–e715. https://doi.org/10.1210/clinem/dgab654.

Sargent, C., Zhou, X., Matthews, R.W., Darwent, D., and Roach, G.D. (2016). Daily rhythms of hunger and satiety in healthy men during one week of sleep restriction and circadian misalignment. Int. J. Environ. Res. Public Health *13*, 170.

Scheer, F.A.J.L., Morris, C.J., and Shea, S.A. (2013). The internal circadian clock increases hunger and appetite in the evening independent of food intake and other behaviors. Obesity *21*, 421–423.

Schommartz, B., Ziegler, D., and Schadewaldt, P. (1998). Significance of diagnostic parameters in [13C] octanoic acid gastric emptying breath tests. Isotopes Environ. Health Stud. *34*, 135–143.

Sensi, S., and Capani, F. (1987). Chronobiological aspects of weight loss in obesity: effects of different meal timing regimens. Chronobiol. Int. *4*, 251–261.

Service, F.J., Molnar, G.D., Rosevear, J.W., Ackerman, E., Gatewood, L.C., and Taylor, W.F. (1970). Mean amplitude of glycemic excursions, a measure of diabetic instability. Diabetes *19*, 644–655.

Sherman, H., Genzer, Y., Cohen, R., Chapnik, N., Madar, Z., and Froy, O. (2012). Timed high-fat diet resets circadian metabolism and prevents obesity. Faseb. J. *26*, 3493–3502.

Speakman, J.R., Yamada, Y., Sagayama, H., Berman, E.S.F., Ainslie, P.N., Andersen, L.F., Anderson, L.J., Arab, L., Baddou, I., Bedu-Addo, K., et al. (2021). A standard calculation methodology for human doubly labeled water studies. Cell Rep. Med. *2*, 100203.

Stote, K.S., Baer, D.J., Spears, K., Paul, D.R., Harris, G.K., Rumpler, W.V., Strycula, P., Najjar, S.S., Ferrucci, L., Ingram, D.K., et al. (2007). A controlled trial of reduced meal frequency without caloric restriction in healthy, normal-weight, middle-aged adults. Am. J. Clin. Nutr. *85*, 981–988.

Sutton, E.F., Beyl, R., Early, K.S., Cefalu, W.T., Ravussin, E., and Peterson, C.M. (2018). Early time-restricted feeding improves insulin sensitivity, blood pressure, and oxidative stress even without weight loss in men with prediabetes. Cell Metab. *27*, 1212–1221.e3.

Tinsley, G.M., Forsse, J.S., Butler, N.K., Paoli, A., Bane, A.A., La Bounty, P.M., Morgan, G.B., and Grandjean, P.W. (2017). Time-restricted feeding in young men performing resistance training: A randomized controlled trial. Eur. J. Sport Sci. *17*, 200–207.

CellPress
OPEN ACCESS

Van Marken Lichtenbelt, W.D., Westerterp, K.R., and Wouters, L. (1994). Deuterium dilution as a method for determining total body water: effect of test protocol and sampling time. Br. J. Nutr. *72*, 491–497.

Versteeg, R.I., Ackermans, M.T., Nederveen, A.J., Fliers, E., Serlie, M.J., and la Fleur, S.E. (2018). Meal timing effects on insulin sensitivity and intrahepatic triglycerides during weight loss. Int. J. Obes. *42*, 156–162.

Wehrens, S.M.T., Christou, S., Isherwood, C., Middleton, B., Gibbs, M.A., Archer, S.N., Skene, D.J., and Johnston, J.D. (2017). Meal timing regulates the human circadian system. Curr. Biol. *27*, 1768–1775.e3.

Westerterp, K.R. (2017). Doubly labelled water assessment of energy expenditure: principle, practice, and promise. Eur. J. Appl. Physiol. *117*, 1277–1285.

Westerterp, K.R., Saris, W.H., van Es, M., and ten Hoor, F. (1986). Use of the doubly labeled water technique in humans during heavy sustained exercise. J. Appl. Physiol. *61*, 2162–2167.

Wilding, J.P.H. (2014). The importance of weight management in type 2 diabetes mellitus. Int. J. Clin. Pract. *68*, 682–691.

Woodie, L.N., Luo, Y., Wayne, M.J., Graff, E.C., Ahmed, B., O'Neill, A.M., and Greene, M.W. (2018). Restricted feeding for 9 h in the active period partially abrogates the detrimental metabolic effects of a Western diet with liquid sugar consumption in mice. Metabolism *82*, 1–13.

Wu, T., Sun, L., ZhuGe, F., Guo, X., Zhao, Z., Tang, R., Chen, Q., Chen, L., Kato, H., and Fu, Z. (2011). Differential roles of breakfast and supper in rats of a daily three-meal schedule upon circadian regulation and physiology. Chronobiol. Int. *28*, 890–903.

Zakrzewski-Fruer, J.K., Gillison, F.B., Katzmarzyk, P.T., Mire, E.F., Broyles, S.T., Champagne, C.M., Chaput, J.P., Denstel, K.D., Fogelholm, M., Hu, G., et al. (2019). Association between breakfast frequency and physical activity and sedentary time: a cross-sectional study in children from 12 countries. BMC Publ. Health *19*, 222–311.

Zarrinpar, A., Chaix, A., Yooseph, S., and Panda, S. (2014). Diet and feeding pattern affect the diurnal dynamics of the gut microbiome. Cell Metab. *20*, 1006–1017.

STAR★METHODS

KEY RESOURCES TABLE

REAGENT or RESOURCE	SOURCE	IDENTIFIER
Biological samples		
Human Blood and Urine Samples	This paper	N/A
Chemicals, peptides, and recombinant proteins		
13C Octanoic Acid	CK Isotopes Ltd, Leicestershire, UK	1-13C, 99%, CLM-293-5
O18-Water 10%	Cortecnet, Voisins-Le-Bretonneux, France	CO5261P1000
Deuterium Oxide, 99.8% - filtered	Cortecnet, Voisins-Le-Bretonneux, France	CD5254P1000
(2-Aminoethyl)-Benzene Sulfonyl Fluoride hydrochloride	AEBSF; Sigma Aldrich, Dorset, UK	11429868001
DiPeptidyl Peptidase IV soluble inhibitor	DPPIV; Merck Millipore (UK) Ltd, Watford, UK	DPP4-010
Protease Inhibitor Cocktail	Sigma Aldrich, Dorset, UK	P2714-1BTL
Critical commercial assays		
Insulin assays	ELISA; Mercodia AB, Uppsala, Sweden	10-1113-01
Diabetes Antigen Control Human (Low/High) for insulin assay	Mercodia AB, Uppsala, Sweden	10-1164-01
Glucose	Konelab 30 selective chemistry analyser (Thermo Scientific, Basingstoke, UK)	N/A
Lipid Profile	Konelab 30 selective chemistry analyser (Thermo Scientific, Basingstoke, UK)	N/A
Human Total GIP kit	MesoScale Discovery (MSD) Rockville, MD, USA	K151RPD1
Human Total Ghrelin kit	EMD Millipore Corporation, St Louis, Missouri 63103, USA	#EZGRT-89K, #EZGRA-89BK
Human Total PYY kit	MesoScale Discovery (MSD) Rockville, MD, USA	K151MPD1
Total GLP-1	MesoScale Discovery (MSD) Rockville, MD, USA	K150JVC
Deposited data		
All data used in figures and supplemental figures are available to download, as the Data S1 file.	N/A	N/A
Software and algorithms		
R version 3.6.3 and version 4.0.5	R Core Team, R Foundation for Statistical Computing, Vienna, Austria	https://www.r-project.org/
Genstat 17 Release 17.1	Lawes Agricultural Trust, VSN international Ltd, Hemel Hempstead, UK	https://www.vsni.co.uk/software/genstat
Actigraph software	Actilife5 Analysis Suite; Actigraph LLC, Pensacola, USA	https://actigraphcorp.com
WinDiets software	Professional Version, Robert Gordon University, Aberdeen, UK, 2017	https://www.rgu.ac.uk/windiets
Quark RMR software	Omnia stand-alone system, COSMED, Rome, Italy	version 1.3, Firmware 4; https://www.cosmed.com/
BodPod system software	COSMED, Rome, Italy	version 5.4.3; https://www.cosmed.com/
dual energy X-ray absorptiometry (DXA) software	Lunar enCORE software; GE Medical Systems Lunar, Madison, USA	version 12.3
CareLink iPro Therapy Management Software (CGMS; web-based system)	Medtronic MiniMed, Northridge, USA	MMT-7340; https://carelink.minimed.eu/ipro/hcp/login.jsf?bhcp=1

(*Continued on next page*)

Continued

REAGENT or RESOURCE	SOURCE	IDENTIFIER
Other		
Stadiometer	Holtain, Crymych, Dyfed, Wales	N/A
Digital Weight Scale	C.M.S. Weighing Equipment, London, UK	DIGI DS-410
Blood Pressure Monitor	Omron Healthcare, Bannockburn, IL	Omron M5-1
Dual energy X-ray absorptiometry (DXA)	Lunar Prodigy/DXA ,GE healthcare, USA	https://www.gehealthcare.com/products/bone-and-metabolic-health/prodigy
BodPod	COSMED, Rome, Italy	#2007A; https://www.cosmed.com/en/products/body-composition/bod-pod
Actigraph (Accelerometers)	Actigraph LLC, Pensacola, USA	GT3X+
Continuous Glucose Monitors (iPro2 digital recorder with Enlite sensors)	Medtronic MiniMed, Northridge, USA	MMT7741 (sensors MMT-7008)
CGMS Docking system	Medtronic MiniMed, Northridge, USA	MMT-7742
Portable glucose monitor	Contour Next BGMS, Ascensia Diabetes Care	https://diabetes.ascensia.com.au/products/contour-next-connected/
RMR with ventilated hood system	Quark RMR, COSMED, Rome, Italy	C09074-01-99 (2012100288 and 2016031059)

RESOURCE AVAILABILITY

Lead contact

Further information and requests for resources and reagents should be directed to and will be fulfilled by the lead contact, Alex Johnstone, (Alex.Johnstone@abdn.ac.uk).

Materials availability

This study did not generate new unique reagents.

Data and code availability

Any additional information required to reanalyze the data reported in this paper is available from the lead contact upon request.

EXPERIMENTAL MODEL AND SUBJECT DETAILS

This study was conducted at the Rowett Institute, University of Aberdeen in accordance with the Helsinki Declaration of 1975 and with ethical approval by the North of Scotland Research Ethics Committee (17/NS/0097). The study was registered at ClinicalTrials.gov under number NCT03305237. Recruitment was by public advertisement of a diet trial for healthy, overweight participants (Body Mass Index (BMI) 27- 40 kg/m^2), aged 18-75 years, using radio adverts, website posts, newsletters, newspapers, public posters and social media. Inclusion criteria specified that all participants should be habitual breakfast eaters and not have existing medical conditions or medication use that could influence their appetite or mood, confirmed during the recruitment process by a medical screening questionnaire. Female participants were included if they were on hormonal contraceptives or were postmenopausal. Exclusion criteria included: heavy smokers (more than 10 cigarettes/day) or heavy alcohol consumers (more than 4 alcohol units/day for male and more than 3 alcohol units/day for female); obesity of endocrine origin; chronic metabolic conditions: diabetes, hepatic disease, gout, kidney, thyroid or coagulation disease; gastrointestinal disorders; psychiatric or eating disorders; gastrointestinal procedure or surgery in the past three months; disorders of swallowing, severe dysphagia to food or pills; pregnancy or lactation; use of implanted or portable electro-mechanical device such as cardiac peacemaker or infusion pump; blood donor in the past 3 months; extremes of chronotypes, sleep patterns or extreme high activity levels. Medication exclusion included: appetite modulator drugs: orlistat, sibutramine, rimonabant; mood disorder medications less than 6 months of use; statins less than 6 months of use; oral antidiabetics, insulin, digoxin, thyroid hormones, antibiotics, steroids or immunosuppressants, recreational substances. All participants underwent a comprehensive screening to determine eligibility including a health status form (to identify known health conditions and medications), measurements of height and weight, assessment of cardiometabolic health (blood pressure, blood hematology and blood chemistry) and measurement of resting metabolic rate (RMR). Questionnaires were used to assess chronotype and sleep and included: Horne-Östberg morningness–eveningness questionnaire (MEQ) (Horne and Östberg, 1976), Munich Chronotype Questionnaire (MCTQ) (Roenneberg et al., 2003, 2007), Pittsburgh Sleep Quality Index (Buysse et al., 1989) and the Epworth Sleepiness Scale (Johns, 1991); and to assess baseline physical activity levels: Baecke physical activity questionnaire (Baecke et al., 1982). All participants gave written, informed consent prior to taking part in the study. A CONSORT flow diagram is provided in Figure 1B. There were 31 participants and 30 completers, with 16 males and 14 females. The mean age of participants was 50.9 yrs (SD 2.1, range 20-79years) (detailed in Table S1). The primary end point was energy balance measured by body weight, which we

considered to be driven by altered energy expenditure since the study provided iso-caloric diets. Secondary end points were subjective appetite control, glycemic control (continuous glucose monitoring and plasma profile), and body composition.

METHOD DETAILS

Study design

This study was conducted as a randomized cross over controlled feeding trial as detailed in Figure 1. Participants were randomly allocated to the sequence of which they were provided the morning loaded (ML) (45%, 35%, 20% of calories at breakfast, lunch and dinner) or evening loaded (EL) (20%, 35%, 45% of calories at breakfast, lunch and dinner) weight loss diets. Each weight loss diet phase was 4 weeks with a lead in screening and baseline phase, and a washout phase between the diets. During the screening (4 days), participants continued to consume their own *ad libitum* diet, and during the baseline diet (B) (4 days) and washout diet (W) (7 days), participants were provided all meals with calories equally distributed between breakfast, lunch and dinner. Dietetic staff of the human nutrition unit supplied all food and drinks consumed during the dietary periods, and participants attended 3 times per week for body weight assessment and food provision. Outcome measures were assessed at the end of each study phase split over 2 test days and included assessments of body composition, RMR and thermic effect of food (TEF), metabolic health parameters (blood pressure (BP), blood lipids, glucose and insulin, gut hormones), gastric emptying and subjective appetite. In addition, during the final week of each study phase, participants underwent several assessments which spanned 3 full days to assess, 24 h blood glucose using continuous glucose monitors (CGMs), physical activity using actigraphy, subjective appetite using VAS and sleep (self-reported record of bedtime, wake time, sleep disturbances and sleep quality). Participants were free to self-select their mealtimes, although they were instructed to maintain the same mealtimes across the duration of the study and to record their mealtimes for 3 consecutive days at the end of each study phase.

Formulation and preparation of the diets

Calories, meal frequency and dietary composition were matched between the ML and EL diets to ensure the only change was due to calorie loading at different times of the day. All food was prepared by the dietetic staff of the human nutrition unit based on a 7 day rotational menu. Menu items on test days were matched across all study phases with adjustments made to maintain the within-diet calorie and macronutrient composition.

During baseline and the washout diets, participants were fed to 1.5 x measured RMR to maintain body weight, provided as three iso-caloric meals/day of which the composition was consistent with standard UK diet composition: 30% fat, 15% protein and 55% carbohydrate. RMR measured during the screening was used to calculate energy requirements for baseline, while RMR measured at the end of baseline, the first weight loss diet (either ML or EL) and the washout phase were used to determine energy requirements for each subsequent phase, first weight loss diet, washout and second weight loss diet respectively. Therefore, energy intake was adjusted throughout the protocol. During the two weight loss diet interventions (ML and EL) participants were fed to 1 x RMR, to achieve caloric deficit with calorie composition across the day and at all meals provided as 35% fat, 30% protein and 35% carbohydrate. The composition of each meal, in terms of energy, fat, carbohydrate and protein, was calculated by using an electronic version of McCance and Widdowson's the composition of foods (Finglas et al., 2014); WinDiets software (Professional Version, Robert Gordon University, Aberdeen, UK, 2017). Participants were encouraged to consume all the daily meals provided. Outside of the prescribed foods and drinks, participants were allowed to consume decaffeinated zero calorie beverages (e.g. water, tea and coffee without milk or sugar or zero calorie soda). Participants recorded daily, the time of eating, when food and drinks consumed from the food provided, on diet-specific forms and they were required to record outside of, or leftover from, the prescribed study meals. For measurement of any foods or drinks each participant received a calibrated kitchen scale (Disc Electronic Kitchen Scale 1036, Salter Housewares, UK), at the start of the study. The final composition of the study diets (based on participant records of extras and leftovers) is provided in Table S2; in brief, subjects consumed ML = 91.7%, EL = 92.5% of food provided. On test days, standard test meal breakfasts were provided. Meals during B and W were identical in composition and foods, with slightly smaller portions if required during W to accommodate a participants lower energy requirement. Similarly, during the ML and EL diets the composition of the meals and foods were identical however portions adjusted to provide 45 versus 20% of energy intake respectively On test day A (fasting assessments of body weight, blood pressure, bone density (DXA) and waist and hip, and fasting and 2 h postprandial blood samples), participants were provided during B and W diets: toasted sandwich (toast, butter, ham, cheese), cereal and milk, fruit juice, yogurt with cream and strawberries, and during ML and EL diets: Toast with butter and chutney, sausage, bacon, chicken, fruit juice and a smoothie (strawberries, passionfruit, cream, milk). During test day B (encompassing assessments of RMR, TEF, Gastric emptying and test day appetite), participants were provided with during the B and W diet: Omelette (eggs and butter), potato waffle, baked beans, toast, orange juice, cereal and milk, and during the ML and EL diets: Omelette (eggs, ham, cheese, mushrooms, spring onions), toast, chutney, and a glass of milk. The baseline and washout diets were identical in composition, fed as three meals a day, of 15% protein, 30% fat, and 55% carbohydrate fed to 1.5 × measured RMR, created to reflect UK average nutrient intake. This menu can be downloaded from Johnstone et al. (2020).

Measurement of anthropometric variables

Measurements of body composition and metabolic rate were performed under standardized fasted conditions as described previously (Johnstone et al., 2005, 2006). Before all the tests, the participants fasted overnight (10 h). During screening height of the

participants was measured to the nearest 0.1 cm using a stadiometer (Holtain Ltd, Crymych, Dyfed, Wales). Body weight was measured at screening, on the morning of all food provision days and on all test days, wearing a previously weighed dressing gown, to the nearest 100g on a digital scale (DIGI DS-410, C.M.S. Weighing Equipment Ltd, London, UK). Body composition was assessed at the end of each dietary phase using the gold standard 4-compartment model as described by Fuller et al. (1992). Body volume and density was measured using air displacement plethysmography with the Bodpod (BodPod Body Composition System; Life Measurement Instruments, COSMED, Concord, CA, USA) and analyzed using the BodPod system's software (version 2.5.2; Norland Corp) as described previously (Johnstone et al., 2006). Total body water (TBW-kg) was measured by deuterium dilution (D2O) according to the Maastricht protocol (Van Marken Lichtenbelt et al., 1994). Briefly, participants were dosed with >0.12 g/kg body water of 99.8% deuterium. Urine samples were collected pre-dose and 6 h and 10 h after the dose for analysis with mass spectrometry (Refer to TDEE section below for further details). Bone mineral mass (Ash-kg) was measured by dual energy X-ray absorptiometry (DXA) (Lunar Prodigy/DXA, GE healthcare, USA) and scans analyzed using the DXA software (Lunar enCORETM software; GE Medical Systems Lunar, Madison, USA). Abdominal (waist) and gluteal (hip) circumferences were taken according to the guidelines of the International Standards for Anthropometric Assessment (Marfell-Jones et al., 2012) and the waist to hip ratio (WHR) calculated.

Total daily energy expenditure by doubly labelled water ($^2H_2$ ^{18}O stable isotopes)

Total daily energy expenditure (TDEE) was measured over the 4 week ML and EL weight loss diets using the Maastricht protocol for the measurement of energy expenditure with doubly labelled water (Westerterp, 2017). Participants were dosed twice with $^2H_2O^{18}$ during each weight loss period (On day 1 and 14 of the ML and EL diets) to measure TDEE over the entire weight loss phases. Prior to dosing, participants provided a spot urine sample to be used as a baseline sample, which was always used to calculate the excess relative abundance of the isotopes after dosing, a standard procedure for repeated doses, described in Westerterp et al., (1986). Following this, participants were provided a pre-prepared dose of $^2H_2O^{18}$. Doses were prepared at University of Maastricht, Netherlands made up to >1.8 g water/kg body water 10% O^{18} (O^{18}-Water 10%, Cortecnet, Voisins-Le-Bretonneux, France, CO5261P1000) and > 0.12 g water/kg body water of 99.8% Deuterium (Deuterium Oxide, 99.8% - filtered, Cortecnet, Voisins-Le-Bretonneux, France, CD5254P1000). The CV for the DLW technique is around 4-8% for the measurement of rCO2 (Westerterp, 2017).

Urine samples were collected pre-dose and then 6 h and 10 h post-dose on the day the dose was consumed, then on days 7, 8, 13 and 14 post doses. The exact time of dosing and sample collection times were recorded. Urine samples were frozen at -20°C until analysis. Urine samples were analyzed at NUTRIM research institute, University of Maastricht, Netherlands, using gas isotope ratio mass spectrometry. Isotopic enrichment of the post-dose urine samples was analyzed relative to background enrichment. Carbon dioxide production rate (rCO_2) was calculated based on newly derived equations (Speakman et al., 2021). rCO_2 was converted to TDEE using standard equations based on the energy equivalents of the diet (Brouwer, 1957). With regard to calculation of energy expenditure, the DLW method measures carbon dioxide production, requiring an estimate of the energy equivalent of carbon dioxide for conversion to energy expenditure. The energy equivalent of carbon dioxide is a function of the substrate mixture being oxidized. As the subjects were in a negative energy balance, and mobilising fat mass, we applied adjustment of body composition, and accounted for diet composition by calculating the energy equivalent from the diet provided (Black et al., 1986).

Resting metabolic rate (RMR) and thermic effect of food (TEF)

RMR was measured over 30 min after a 10 h overnight fast by indirect calorimetry using a ventilated hood system (Quark RMR, COSMED, Rome, Italy). The first 5 min of the measurement were excluded and the RMR was calculated using a moving average to determine the 15 min period with the lowest CV. RMR was calculated using the equations of Elia & Livesey (Elia and Livesey, 1992). The system was calibrated prior to all measurements with a standardized 3 L syringe and gas concentration using a two-point calibration procedure using room air and standardized calibration gas. The thermic effect of food (TEF) was assessed after the consumption of a test breakfast over a 6 h period for 10 min every 30 min. The post-prandial increase in energy expenditure was analyzed as area under the curve (AUC) using the trapezoidal rule to calculate TEF (Reed and Hill, 1996). Throughout the measurement, participants lay on a bed and were instructed to lie still but not to fall asleep.

Assessment of gastric emptying

Gastric emptying was measured in the morning following a breakfast test meal using the ^{13}C octanoic acid breath test (^{13}C-OA) (Ghoos et al., 1993). 100μL of ^{13}C OA (1-13C, 99%, CLM-293-5, CK Isotopes Ltd, Leicestershire, UK) was added to a test meal omelet using a calibrated pipette (3120000.054, Eppendorf UK Limited, Stevenage, UK). Eighteen sets of breath samples were collected into evacuated exetainer breath vials (Labco Limited, Lampeter, UK), pre-meal then every 15 min for the first 2.5 h followed by every 30 min until 6 h post-meal. ^{13}C enrichment of breath samples were measured by isotope ratio mass spectrometry (IRMS; Roboprep G, Tracermass; Europa Scientific, Crewe, UK) to determine the ratio of $^{13}CO_2$:$^{12}CO_2$ corrected for baseline $^{13}CO_2$ enrichment. The standard deviation of the replicate samples was < 0.0004 (CV>0.04). All modelling (curve-fitting) was done in R (version 4.0.5) using the nonlinear least square's function. Rate of CO_2 production (P, mmol/min) per participant was estimated from body surface area using the height-weight formula defined by Haycock et al. (1978). The data was fitted to the $mk\beta$ model presented in Ghoos et al. (1993): $y = m(1 - e^{-kt})^{\beta}$, where y is the cumulative percentage dose of $^{13}CO_2$ recovered at time-point t (mins), and m, k, and $ß$ are constants with m being the total cumulative $^{13}CO_2$ recovery when time is infinite. The model was used to calculate the standard time-based parameters as described by Ghoos et al. (1993): half time (T_{half}), the time point at which 50% of the total excretion of

$^{13}CO_2$ in the breath has been recovered, lag time (t_{lag}), the time point at which the rate of excretion reaches its maximum; as well as the parameters proposed by Schommartz et al. (1998): latency (t_{lat}), describing the initial delay (latency) in $^{13}CO_2$ excretion, and ascension time (t_{asc}), a measure for the length of time during which excretion is rapid, i.e. when the cumulative curve is ascending.

Assessment of subjective appetite

Appetite was assessed over 3 full days and on test days by use of pen and paper 100mm visual analogue scales (VAS) as previously validated (Flint et al., 2000). On test days the VAS was completed before eating and every 30 min, thereafter, as described previously. During the 3 day assessments, participants were instructed to compete the questionnaire hourly across their entire waking day starting from when they first awoke to immediately prior to sleep. The questionnaires contained six questions related to motivation to eat: hunger, fullness, prospective consumption (quantity), desire to eat, thirst and preoccupation with thoughts of food. The scales were recording from, for example, ‘‘not at all hungry’’ to ‘‘as hungry as I ever felt’’ so that higher scores indicated more intense subjective sensations. A summary measure of subjective appetite was calculated from the responses to the questionnaires using the formula:

Appetite score = [hunger + (100 - fullness) + prospective consumption + desire to eat]/4 (Anderson et al., 2002).

Metabolic profile and glucose homeostasis

Blood pressure was measured before breakfast in a semi reclined position during screening and at the end of each dietary intervention period with the use of an automated system (Omron M5-1; Omron Healthcare Inc, Bannockburn, IL) with the average of 3 measures recorded. The following blood tests were performed based on blood collected fasting and 2 h postprandial by standard venipuncture: plasma glucose, plasma insulin, lipid panel, and gut peptides (Ghrelin, GIP, PYY, GLP-1). 160μL of a previously prepared inhibitor mix containing (2-Aminoethyl)-BenzeneSulfonyl Fluoride hydrochloride (AEBSF; Roche Diagnostics, Basel, Switzerland), Di-Peptidyl Peptidase IV soluble inhibitor (DPPIV; Merck Millipore, Watford, UK) and Protease Inhibitor Cocktail (Sigma Aldrich, Dorset, UK) was added to the gut hormone blood samples immediately post sample collection. Samples were centrifuged at 3000rpm for 15 min at 4°C. Plasma aliquots were stored at −70°C until analysis. Glucose and lipid analysis was undertaken using KONE calorimetric assays, in a KONELAB 30 selective chemistry analyzer (Thermo Scientific, Basingstoke, UK) performed at the University of Aberdeen. Insulin was measured using ELISA with commercially available kits (Mercodia, Uppsala, Sweden #10-1113-01). The fasted glucose & insulin results were applied to the homeostatic model assessment (Matthews et al., 1985) to calculate insulin resistance (HOMA-IR), beta-cell function (HOMA-βcell) and the insulin to glucose ratio (IGR). Gut peptides were analyzed at the Core Biochemical Assay Laboratory of the National Institute for Health Research Cambridge Biomedical Research Centre using commercially available kits. Total Ghrelin was analyzed using sandwich ELISA using a Human Total Ghrelin kit (EMD Millipore Corporation, St Louis, Missouri 63103, USA, #EZGRT-89K, #EZGRA-89BK). Total GIP, GLP-1 and PYY were measured by sandwich immunoassay using MesoScale Discovery kits (Rockville, MD, USA) (GIP: K151RPD1, GLP-1: K150JVC, PYY- K151MPD1) and analyzed using electrochemiluminescence on a MesoScale Discovery (MSD) s600 instrument.

Continuous glucose monitoring

Continuous glucose monitors (iPro2, Medtronic MiniMed Inc, Northridge, USA) were used to measure interstitial glucose profiles for ≥72 h at the end of each study phase. Glucose sensors (Enlite Glucose Sensors, Medtronic) were inserted subcutaneously on the abdomen with measurements taken every 10 s and average glucose values stored in the attached Ipro2 monitor for each 5 min period. Finger-prick glucose assessments were taken by the participants 4 times per day with a small portable glucose monitor (Contour Next BGMS, Ascensia Diabetes Care) to calibrate the CGMs. All data was downloaded from the devices at the end of the 3 days of measurements. Glucose parameters were calculated over 24 h and 2 h postprandially, for the following parameters: mean, maximum, minimum, AUC, incremental AUC (iAUC), SD, and mean amplitude of glycemic excursions (MAGE). MAGE is a commonly used comprehensive index to determine intraday glycemic variability and was calculated as the mean of the differences between consecutive peaks and nadirs greater than one SD of mean daily glucose (Monnier et al., 2008; Service et al., 1970).

Sleep record

Participants were asked to complete a brief record, and to answer questions related to their sleep for 3 consecutive days at the end of each dietary phase. Participants were asked to complete these immediately upon waking. The record included: Bed-time, Sleep-time, estimated sleep latency (duration from attempting to sleep to actual sleep), wake time, arise time, number and estimated duration of night time wakening’s and a rating of sleep quality (9 point scale from 1 best sleep ever to 9 worst sleep ever).

Physical activity

Physical activity was measured using triaxial accelerometry (ActiGraph, LLC, Pensacola, USA), worn for 3 days in the final week of each study phase. The devices were worn on the left anterior hip and participants were instructed to put them on as soon as they woke up and remove only while showering or swimming or before bed. Participants recorded the actual times they put on and removed the monitors and any reasons for their removal. Data was downloaded and analyzed on the Actilife analysis platform (Actilife5 Analysis Suite; Actigraph LLC, Pensacola, USA).

QUANTIFICATION AND STATISTICAL ANALYSIS

Power analysis

Sample size calculation was based on the primary endpoint, difference in body weight at the end of the 4 week diets. We aimed to be able to detect differences of 1–1.2 kg with a SD between groups of approximately 1.4 kg as reported previously (Jakubowicz et al., 2013). To obtain 90% power, with a 5% significance level we required 30 subjects to complete both of the 4 week arms of the weight loss diets. Details on the statistical parameters can be found in the figures and legends.

Statistical analysis

Randomization for diet order was conducted by Computer-generated random numbers to assign the participants to first receive either the ML or EL diet. Statistical analysis was completed using R (R Foundation for Statistical Computing, Vienna, Austria. URL https://www.R-project.org/) and Genstat 17 Release 17.1 (Lawes Agricultural Trust, VSN international Ltd, Hemel Hempstead, UK). Normality was assessed by inspection of histograms of variables and residuals of the fitted models. If variables showed some indications of skewness, analysis was repeated on a log scale to confirm that conclusions remained unchanged. Data on energy intake, energy expenditure, body weight and composition and gastric emptying were analyzed by hierarchical ANOVA (linear mixed model for balanced data), with participant as blocking factors (random effect) and diet as treatment term (fixed effect) and, depending on the specific response, order of diet within study, week of diet within study, clock time for time x diet effects, day within diet, or participant characteristics, as additional fixed effects. Blood metabolites were log transformed prior to being analyzed by hierarchical ANOVA. When the effect of diet was significant ($p < 0.05$), means were compared with Tukey adjusted post hoc t-tests as a multiple comparison correction; applied for primary and secondary outcomes. Subjective appetite ratings were analyzed by mixed models, with random effect terms for participant, diet, day within the diet (for 3 day VAS), time and their interactions, and fixed effect terms for diet, time of day, and their interactions. Continuous glucose monitor parameters were analyzed using by mixed models, with random effect terms for participant, diet, day within the diet and their interactions and fixed effect terms for diet, day and day by diet interaction. All results are reported as estimated marginal mean ± SED (SE of the difference, e.g. between diets) or Mean ± SEM (SE of the mean). There were no significant order effects for outcomes.

Cell Metabolism, Volume *34*

Supplemental information

Timing of daily calorie loading affects appetite and hunger responses without changes in energy metabolism in healthy subjects with obesity

Leonie C. Ruddick-Collins, Peter J. Morgan, Claire L. Fyfe, Joao A.N. Filipe, Graham W. Horgan, Klaas R. Westerterp, Jonathan D. Johnston, and Alexandra M. Johnstone

Supplemental Figure 1:

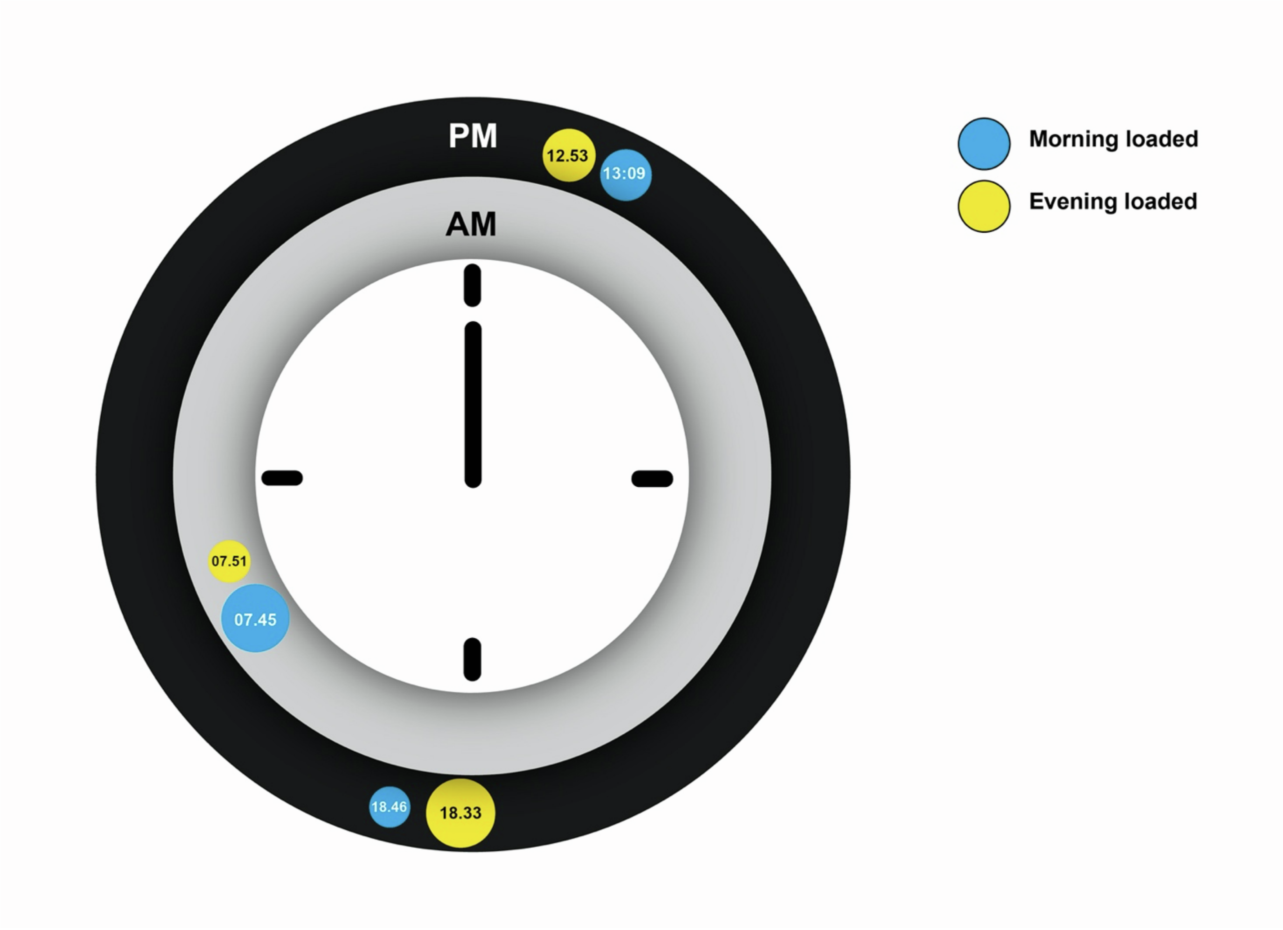

Supplemental Figure 1: Average recorded subjects' mealtime for the meals on the morning loaded (ML, blue circles) and evening loaded (EL, yellow) diets, where the size of the circle reflects the size of the meal, related to Figure 1.

Supplemental Figure 2:

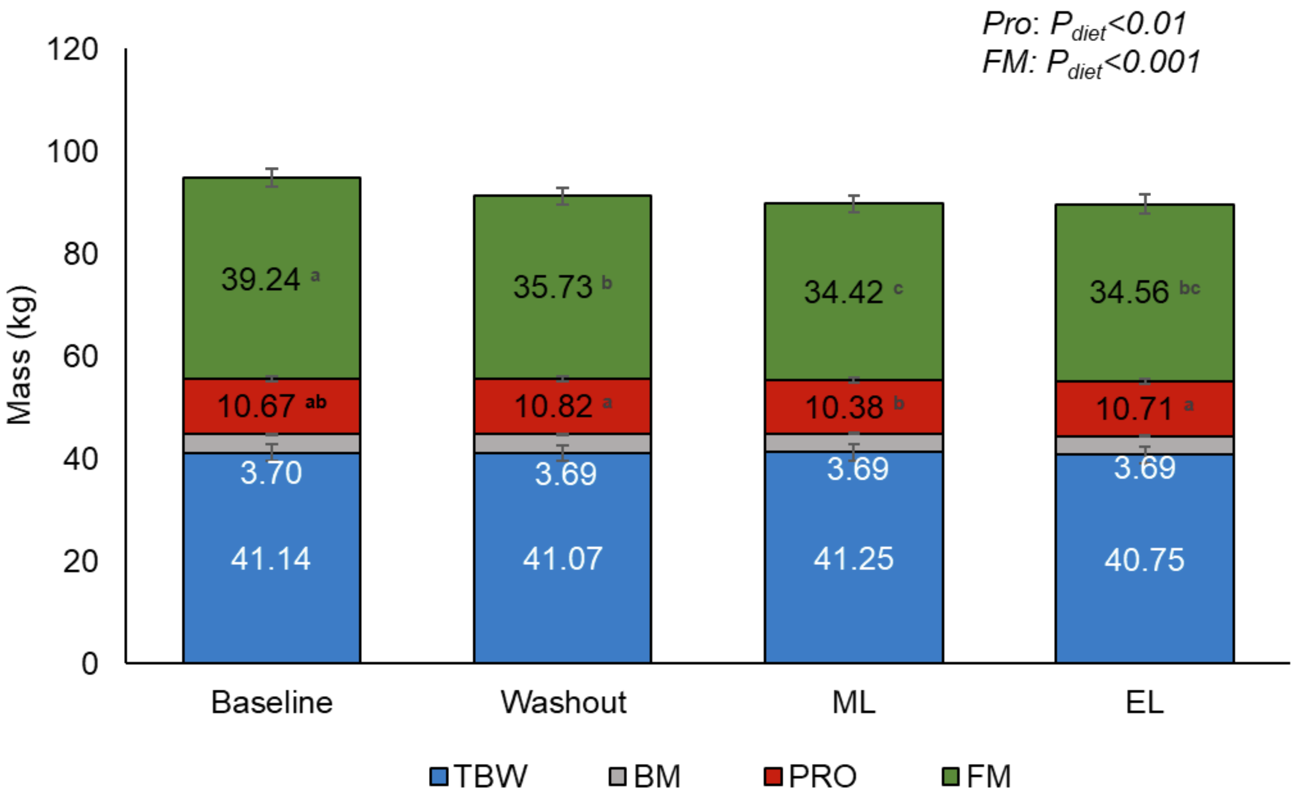

Supplemental Figure 2: Body composition measured with the 4-compartment model, related to Figure 2. Body volume (densitometry) derived from the BODPOD, total body mineral derived from dual energy x-ray absorptiometry (DXA) and total body water (TBW) from deuterium isotope dilution. Participants had significantly lower body weight following both the ML and EL diets compared to B and W, but with no difference between the two weight loss diets. FM was significantly lower at the end of the ML and EL diets compared to B, but there was no significant difference between the weight loss diets. There were no significant differences in bone mineral or TBW between any of the diets. Protein mass was significantly lower after the ML diet compared to EL diet and W, but it was not lower than B.

B: baseline, BM: bone mineral, EL: evening loaded, FM: fat mass, ML: morning loaded, PRO: protein mass, W: washout

Supplemental Figure 3:

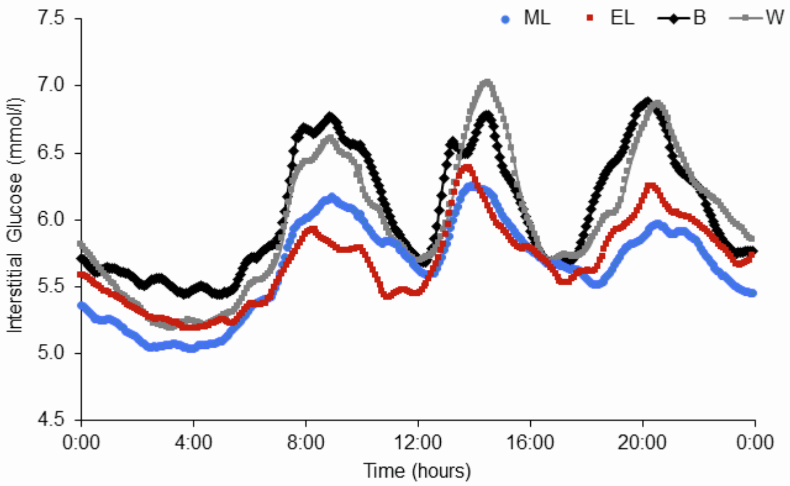

Supplemental Figure 3: Group mean continuous glucose monitoring for all diet periods, related to Figure 5

Supplemental Table 1: Pre-intervention characteristics of overweight adults who completed the dietary protocol, related to Figure 4.

	Males (n = 16)		Females (n = 14)		All (n = 30)	
Age (y)	51.0 ± 3.0	(27 - 70)	50.8 ± 3.2	(29 - 67)	50.9 ± 2.1	(27 - 70)
Height (m)	1.78 ± 0.01	(1.67 - 1.90)	1.63 ± 0.02	(1.50 - 1.73)	1.71 ± 0.02	(1.50 - 1.90)
Weight (kg)	105.6 ± 3.7	(86.1 - 135.3)	83.7 ± 2.6	(73.6 - 109.0)	95.4 ± 3.1	(73.6 - 135.3)
BMI (kg/m^2)	33.5 ± 1.2	(27.3 - 41.8)	31.3 ± 0.7	(28.0 - 38.6)	32.5 ± 0.7	(27.3- 41.8)
RMR (kcal)	2112 ± 71	(1814 – 2894)	1534 ± 48	(1153 – 1881)	1842 ± 69	(1153 – 2894)
SBP (mmHg)	138 ± 3	(111 – 165)	128 ± 4	(103 – 156)	133 ± 3	(103 – 165)
DBP (mmHg)	88 ± 2	(75 – 104)	84 ± 2	(70 – 97)	86 ± 2	(70 – 104)
Pulse (beats/min)	62 ± 2	(51 – 78)	65 ± 3	(52 – 82)	63 ± 2	(51 – 82)
HbA1c (mmol/mol)	36.1 ± 1.0	(29 – 42)	36.3 ± 1.0	(29 – 45)	36.2 ± 0.7	(29 – 45)
Chronotype (MEQ)[a]	3.8 ± 0.3	(2 – 5)	3.6 ± 0.3	(2 – 5)	3.7 ± 0.2	(2 – 5)
PSQI score[b]	5.3 ± 0.8	(1 – 12)	5.2 ± 0.7	(2 – 11)	5.2 ± 0.5	(1 – 12)

Data are expressed as mean ± SEM; range in parentheses

[a]MEQ: Morning -Evening questionnaire (Horne and Östberg, 1976) Chronotype type range: 1 – 5. (1 = definitely evening to 5 = definitely morning).

[b]PSQI: Pittsburgh sleep quality index (Buysse et al., 1989). Range: 0–28. Score ≤14 normal >14 indicative of insomnia.

DBP: Diastolic blood pressure, EL: evening loaded, ML: morning loaded, RMR: resting metabolic rate, SBP: Systolic blood pressure

Supplemental Table 2: Macronutrient composition of the diets consumed during the study dietary periods, as described in the STAR methods section.

Diet	Energy (kcal/d)	Fat (%)	Protein (%)	CHO (%)	Alcohol (%)	Fiber (%)
Baseline	2658[a]	30.0[b]	15.1 [b]	53.0[b]	0.2[b]	1.8[b]
Washout	2472[c]	30.0[b]	15.1 [b]	53.0[b]	0.1[b]	1.8[b]
ML	1736[d]	34.7[a]	29.7[c]	34.2[c]	0.1[b]	1.3[c]
EL	1749[d]	34.8[a]	29.8[c]	34.0[c]	0.1[b]	1.4[c]
SED	72.7	0.58	0.39	0.74	0.61	0.044
P	< 0.001	< 0.001	< 0.001	< 0.001	< 0.001	< 0.001

Data are expressed as means, *n*=30.

Analyzed for diet effect by ANOVA, means in the same row not sharing a superscript are significantly different ($P<0.05$). SED is based on within-volunteer spread.

CHO: carbohydrate, EL: evening loaded, ML: morning loaded

Supplemental Table 3: Meal and Sleep times throughout the study dietary periods, as decribed in the STAR methods section.

	Baseline	Washout	ML	EL	SED	P
Mealtime						
Breakfast (HH:MM)	08:07^{a}	07:46^{b}	07:43^{b}	07:52^{ab}	00:07	0.011
Lunch (HH:MM)	13:00	13:06	13:10	12:58	00:07	0.251
Dinner (HH:MM)	18:27^{a}	18:44^{ab}	18:51^{b}	18:32^{a}	00:06	0.002
Duration of feeding (HH:MM)	10:20^{a}	10:56^{bc}	11:05^{b}	10:39^{ac}	00:09	< 0.001
Sleep parameters						
Wake time (HH:MM)	06:00	06:07	06:03	06:04	00:05	0.675
Arise time (HH:MM)	06:14	06:26	06:21	06:26	00:06	0.201
Bed time (HH:MM)	21:57^{a}	22:09^{ab}	22:16^{b}	22:13^{b}	00:06	0.016
Sleep time (HH:MM)	22:16^{a}	22:35^{b}	22:34^{b}	22:32^{b}	00:05	0.005
Sleep latency	11:87^{a}	8.34^{b}	7.68^{b}	9.96^{ab}	0.089	< 0.001
Sleep disturbances	1.23^{a}	0.72^{b}	0.64^{b}	0.69^{b}	0.046	< 0.001
Sleep quality	4.56	4.11	4.28	4.15	0.21	0.13
Attempted sleep duration (HH:MM)	07:43	07:32	07:30	07:32	00:07	0.265
Actual sleep duration (HH:MM)	07:06	07:08	07:08	07:02	00:08	0.888

Analyzed for diet effect by ANOVA, means in the same row not sharing a superscript are significantly different ($P<0.05$). SED is based on within-volunteer spread. Sleep latency and sleep disturbance the reported SED is actually SE of the ratio between diets due to using log transformed values. Sleep quality score range: 1-9, 1 = best, 9 = worst. Actual sleep duration based on difference between sleep time and wake time subtracting away sleep latency and participant estimated duration of night-time awakenings. Sleep disturbances refers to the number of times an individual awoke during the night.

EL: evening loaded, ML: morning loaded, SED: standard error of the difference; averaged over pairs when comparing more than two treatments

Supplemental Table 4: Gastric emptying results, related to Figure 4

	Baseline	Washout	ML	EL	SED	P
T_{asc} (h:mm)	3:57 ^{ab}	3:43 ^{a}	4:38 ^{b}	2:50^{c}	0:17	<0.001
T_{half} (h:mm)	6:08^{a}	5:56 ^{a}	6:25^{a}	4:07^{b}	0:20	<0.001
T_{lag} (h:mm)	4:37 ^{a}	4:29 ^{a}	4:23^{a}	2:57^{b}	0:14	<0.001
T_{lat} (h:mm)	2:11^{a}	2:12 ^{a}	1:46^{b}	1:17^{c}	0:10	<0.001

Gastric emptying measured using the ^{13C} octanoic acid breath test.

Analyzed for diet effect by ANOVA, means in the same row not sharing a superscript are significantly different (P<0.05). SED is based on within-volunteer spread.

The data was fitted to the $mk\beta$ model presented in Ghoos et al (Ghoos et al., 1993): $y = m(1 - e^{-kt})^{\beta}$, where y is the cumulative percentage dose of $^{13}CO_2$ recovered at time-point t (mins), and $m, k,$ and β are constants with m being the total cumulative $^{13}CO_2$ recovery when time is infinite. All modelling (curve-fitting) was done in R (version 4.0.5) using the nonlinear least square's function.

EL: evening loaded, ML: morning loaded, SED: standard error of the difference; averaged over pairs when comparing more than two treatments, T_{asc}: the length of time during which $^{13}CO_2$ excretion in the breath is rapid i.e. when the cumulative curve is ascending, T_{half}: timepoint at which 50% of the total excretion of $^{13}CO_2$ in the breath has been recovered, T_{lag}: timepoint at which $^{13}CO_2$ excretion rate in the breath is at its maximum, T_{lat}: initial delay or latency of $^{13}CO_2$ excretion in the breath

Supplemental Table 5. Glucose parameters calculated from continous glucose monitors, related to Figure 5

	Baseline	Washout	ML	EL	SED	P_{diet}	$P_{wt.loss}$	$P_{diet*wt.loss}$
MAGE (mmol/L)	3.03	3.02	2.56	2.32	0.19	<0.001	<0.001	0.389
Mean (mmol/L)								
Whole day	6.13	5.96	5.62	5.67	0.10	<0.001	<0.001	0.598
Breakfast	7.06	6.80	6.34	6.10	0.15	<0.001	<0.001	0.213
Lunch	6.92	6.86	6.41	6.31	0.18	0.001	<0.001	0.860
Dinner	6.99	9.72	6.07	6.26	0.16	<0.001	<0.001	0.410
SD (mmol/L)								
Whole day	0.96	0.96	0.72	0.71	0.06	<0.001	<0.001	0.717
Breakfast	0.66	0.69	0.64	0.60	0.06	0.420	0.324	0.396
Lunch	0.76	0.81	0.59	0.66	0.08	0.018	0.003	0.586
Dinner	0.74	0.75	0.53	0.52	0.07	0.003	<0.001	0.408
AUC (mmol • min/L)								
Whole day	8793	8559	8053	8133	140	<0.001	<0.001	0.589
Breakfast	815	785	732	704	18	<0.001	<0.001	0.224
Lunch	799	792	740	729	21	0.001	<0.001	0.859
Dinner	807	775	700	721	18	<0.001	<0.001	0.423
iAUC (mmol • min/L)								
Whole day	1079	849	684	581	106	0.005	<0.001	0.485
Breakfast	120	127	90	71	11	<0.001	<0.001	0.050
Lunch	152	153	103	108	17	0.003	<0.001	0.824
Dinner	125	135	86	101	17	0.018	0.007	0.242
Minimum (mmol/L)								
Whole day	4.55	4.51	4.47	4.48	0.15	0.981	0.683	0.994
Breakfast	5.93	5.63	5.41	5.24	0.12	<0.001	<0.001	0.052
Lunch	5.52	5.43	5.43	5.27	0.13	0.429	0.346	0.391
Dinner	5.71	5.45	5.23	5.32	0.11	0.001	<0.001	0.103
Maximum (mmol/L)								
Whole day	8.70	8.66	7.81	7.86	0.23	<0.001	<0.001	0.814
Breakfast	7.97	7.77	7.32	6.99	0.21	<0.001	<0.001	0.286
Lunch	7.87	7.90	7.22	7.25	0.25	0.003	<0.001	0.952
Dinner	7.96	7.76	6.86	6.99	0.23	<0.001	<0.001	0.824

Glucose indices measured with continuous glucose monitors during a 2 to 3-day period at the end of each dietary phase. Whole day represents 24-hour measures between 00:00 until 00:00 the

following day. Breakfast, lunch and dinner measurements taken from the 2-hour period following the timing of meals recorded by participants. Analyzed using linear mixed models.

EL: evening loaded, MAGE: mean amplitude of glycaemic excursions, ML: morning loaded, SED: standard error of the difference; averaged over pairs when comparing more than two treatments

Supplemental Table 6. Fasting and 2-hour plasma glucose and lipids, as described in STAR methods.

	Time (h)	Baseline	Washout	ML	EL	SED_{diet}	$SED_{time*diet}$	P_{diet}^{2}	$P_{time*diet}^{2}$
Cholesterol (mmol/L)	0	5.50^{a}	4.86^{b}	4.92^{bc}	4.94^{bc}				
	2	5.63^{a}	5.00^{c}	5.00^{bc}	4.84^{bc}				
	ALL	5.56^{a}	4.93^{b}	4.96^{b}	4.89^{b}	0.14	0.15	<0.001	0.043
HDL-Cholesterol (mmol/L)	0	1.28	1.17	1.27	1.22				
	2	1.24	1.17	1.26	1.22				
	ALL	1.26^{a}	1.17^{b}	1.26^{a}	1.22^{ab}	0.36	0.04	0.037	0.415
LDL-Cholesterol (mmol/L)	0	3.26	2.84	2.97	2.90				
	2	3.23	2.83	2.94	2.94				
	ALL	3.24^{a}	2.84^{b}	2.95^{b}	2.92^{b}	0.11	0.11	0.002	0.390
TC-HDL Cholesterol Ratio	0	4.71^{a}	4.35^{bd}	4.04^{c}	4.23^{bcd}				
	2	4.93^{a}	4.54^{ab}	4.18^{d}	4.13^{cd}				
	ALL	4.82^{a}	4.45^{ab}	4.11^{b}	4.18^{b}	0.21	0.22	0.002	0.003
LDL-HDL Cholesterol Ratio	0	2.82	2.54	2.45	2.48				
	2	2.84	2.57	2.45	2.53				
	ALL	2.83	2.55	2.45	2.51	0.14	0.15	0.081	0.583
NEFA (mmol/L)	0	0.59	0.60	0.75	0.67				
	2	0.21	0.17	0.25	0.25				
	ALL	0.40^{a}	0.38^{a}	0.50^{b}	0.46^{b}	0.03	0.04	<0.001	0.102
Triglycerides (mmol/L)	0	1.77^{a}	1.38^{c}	0.97^{d}	0.99^{d}				
	2	2.35^{b}	1.96^{a}	1.24^{c}	1.11^{d}				
	ALL	2.06^{a}	1.67^{b}	1.11^{c}	1.05^{c}	0.10	0.11	<0.001	<0.001
Glucose (mmol/L)	0	5.70	5.30	5.27	5.30				
	2	5.49	5.17	4.73	4.55				
	ALL	5.60^{a}	5.24^{b}	5.00^{bc}	4.92^{c}	0.12	0.17	<0.001	0.132

Blood metabolites were log transformed prior to being analyzed by hierarchical ANOVA. Means for each metabolite for 0 h and 2 h measures not sharing a superscript are significantly different ($P<0.05$). Means within each row specified *ALL* not sharing a superscript are significantly different ($P<0.05$).

EL: evening loaded, ML: morning loaded, NEFA: non-esterified fatty acids, SED: standard error of the difference; averaged over pairs when comparing more than two treatments, TC: total cholesterol
